# Designing MOF-Cellulose Bio-Aerogels for Electromagnetic Management and Fire-Acoustic Safety

**DOI:** 10.34133/research.1111

**Published:** 2026-02-06

**Authors:** Jinhu Hu, Jierui Ye, Pooya Jafari, Boyou Hou, Jinfeng Li, Toan Dihn, Jiao Liu, Pan Chen, Mingliang Ma, Min Hong, Ye-Tang Pan, Pingan Song

**Affiliations:** ^1^ School of Materials Science and Engineering, Beijing Institute of Technology, Beijing 100081, China.; ^2^ National Engineering Research Center of Flame Retardant Materials, School of Materials Science and Engineering, Beijing Institute of Technology, Beijing 100081, China.; ^3^ Centre for Future Materials, School of Agriculture and Environmental Science, University of Southern Queensland, Springfield, Queensland 4300, Australia.; ^4^ Electronic Information School, Wuhan University, Wuhan 430072, Hubei, China.; ^5^ Centre for Future Materials, School of Engineering, University of Southern Queensland, Springfield, Queensland 4300, Australia.; ^6^ School of Civil Engineering, Qingdao University of Technology, Qingdao 266520, Shandong, China.

## Abstract

Lightweight multifunctional aerogels hold great promise in applications, e.g., electromagnetic microwave absorption, thermal insulation, and acoustic damping. However, conventional aerogels often suffer from limited functionalities, complicated manufacturing, and poor sustainability. Metal-organic frameworks (MOFs), with tunable porosity and abundant active sites, offer a compelling route to high-performance multifunctional aerogels, but it has remained a grand challenge to develop sustainable multifunctional MOF-based aerogels. Here, we report a sustainable multifunctional bio-aerogel (Ni-CCA) by integrating hierarchical scale-like topological Ni-MOF-NH_2_ with cellulose through simple pretreatment using deep eutectic solvent followed by stepwise assembly–carbonization. The resulting aerogel features an ultralow density and a 3-dimensional layered porous structure. With 5 wt.% filler loading, Ni-CCA achieves a minimum reflection loss (RL_min_) of −53.47 dB and an effective absorption bandwidth of 4.42 GHz, along with a radar cross-section suppression of 27.90 dB·m^2^. Additionally, Ni-CCA shows enhanced flame retardancy (64.3% reduction in peak heat release), low thermal conductivity [33.3 mW/(m·K)] and improved acoustic damping (NRC of 0.31, 15 to 23 dB attenuation). The multifunctionalities of this bio-aerogel stem from its hierarchical architecture and synergistic loss mechanisms, offering a promising strategy for creating the next generation of lightweight multifunctional protective materials.

## Introduction

With the rapid advancement of electronic information technology, aerospace, military equipment, building energy conservation, and environmental governance, materials are increasingly required to operate in complex and diverse environments, posing unprecedented challenges to their overall performance [1–4]. In particular, in typical application scenarios such as high-performance electromagnetic microwave (EMW) absorption, fire protection, thermal infrared stealth, and noise control, conventional functional materials often possess only a single capability, making them inadequate for meeting practical demands under multi-physical-field coupling conditions [5,6]. Therefore, the development of multifunctional material systems that simultaneously offer EMW absorption, flame retardancy, and thermal insulation—while maintaining light weight, high stability, and environmental sustainability—has become a critical focus in current materials science research [7–9].

As a class of promising multifunctional platforms, aerogels are widely regarded as ideal platforms for multifunctional integration due to their ultralow density, high specific surface area, hierarchical porous structure, and excellent structural design flexibility [10,11]. By tailoring the skeleton structure and composition, aerogels can exhibit remarkable advantages in EMW absorption, thermal shielding, and acoustic damping [12,13]. However, different categories of aerogels still face inherent limitations. For example, silica aerogels possess exceptional thermal insulation performance but suffer from high brittleness, poor processability, and energy-intensive fabrication [14]. In contrast, polymer-based aerogels (such as polyimide or polyurethane aerogels) offer improved mechanical resilience but rely on petroleum-derived feedstocks, are nonbiodegradable, and therefore deviate from the trend toward sustainable and green manufacturing [15]. Against this backdrop, natural cellulose, with its abundant availability, environmental friendliness, and renewability, has garnered growing research interest. As the most abundant organic polymer in nature, cellulose exhibits excellent biocompatibility, film-forming ability, and mechanical strength. Its molecular structure is rich in hydroxyl groups, endowing it with strong potential for chemical modification [16]. Through nanofiberization and functionalization, cellulose can be fabricated into porous aerogel networks with good thermal stability and mechanical flexibility [17,18]. Nevertheless, due to its inherently low electrical conductivity and weak magnetic response, pristine cellulose alone is insufficient to achieve effective broadband EMW absorption. This limitation underscores the urgent need to incorporate functional nanocomponents through synergistic assembly to break through its performance bottleneck.

To overcome the functional limitations of cellulose aerogels, metal-organic frameworks (MOFs) have emerged as particularly promising candidates due to their highly tailorable structures and functional tunability, offering an ideal strategy for enhancing the performance of biomass-based aerogels [19]. MOFs possess high specific surface areas, periodic pore networks, and tunable metal–ligand coordination, enabling precise modulation of dielectric polarization, magnetic loss, acoustic scattering, and thermal transport behaviors within composite systems [20]. Recent studies have demonstrated that MOF-derived materials exhibit considerable potential in EMW absorption, thermal insulation, acoustic regulation, and flame retardancy. Cao et al. [21] fabricated a ZIF-67-derived 3-dimensional (3D) porous aerogel (Co-rGO-pvp) with outstanding EMW absorption properties. This material achieved a strong reflection loss (RL) of −40.57 dB at 2.0 mm thickness and an effective absorption bandwidth (EAB) of 7.12 GHz at 2.5 mm. Fan et al. [22] developed a hierarchically layered and tortuous porous aerogel (PAN@ZIF-8-Kevlar), which exhibited a high sound absorption coefficient of 0.99 at 500 Hz and a thermal conductivity close to that of air [24.6 mW/(m·K)]. Our team has also systematically explored the synergistic effects of MOFs in polymer flame-retardant systems [23]. By designing MOF derivatives with unique nanostructures, such as hollow nanocages [24,25], porous nanosheets [26], and nuclear pore frameworks [27], the flame retardancy of epoxy resins is markedly improved. Studies have shown that transition metals in MOFs (e.g., Co and Ni) can catalyze the formation of compact char layers during combustion, while their porous structures can efficiently adsorb toxic gases and smoke particles. However, despite the remarkable progress achieved in the development of MOF-based materials for single-function applications, current research remains limited in several key aspects. Most studies focus on one specific property of MOFs, while systematic investigations into the integrated design, interfacial compatibility, and synergistic multifunctionality between MOFs and biomass matrices are still lacking. In particular, there is a notable absence of studies that simultaneously achieve EMW absorption, flame retardancy, thermal insulation, and acoustic damping within a sustainable, low-filler-loading MOF-cellulose aerogel system. Existing work has yet to fully address the multifunctional requirements demanded by practical applications, nor has it provided a deep understanding of interfacial interactions, energy transfer, and dissipation mechanisms within the composite systems. Therefore, developing a composite aerogel that effectively combines the functional attributes of MOFs with the sustainability advantages of cellulose, while achieving synergistic enhancement across multiple properties, holds substantial scientific relevance and application potential.

This work proposes a novel 3D layered composite aerogel (Ni-CCA) based on unique scale-like Ni-MOF-NH_2_ and modified cellulose. The strategy involves pretreating cellulose with a P/N/S ternary deep eutectic solvent to introduce various polar groups, enhancing the reactivity and polarization performance of the cellulose framework. Subsequently, Ni^2+^ and 2-aminoterephthalic acid (2-ATA) are coordinated to self-assemble into layered Ni-MOF-NH_2_, which are embedded into the cellulose network to form a precursor gel. Through a 2-step carbonization process, the scale-like flakes are converted into dispersed Ni_2_P nanoparticles embedded within a heteroatom-doped carbon skeleton, resulting in an aerogel system with heterointerfaces, hierarchical porosity and synergistic magnetic-electrical conductivity. Systematic testing shows that the Ni-CCA achieves excellent EMW absorption capability at extremely low filler loading (5 wt.%). Additionally, it exhibits a low room-temperature thermal conductivity of 33.3 mW/(m·K), thereby markedly enhancing thermal insulation and infrared stealth performance. The aerogel maintains its structural integrity under high-temperature conditions, reflecting strong flame-retardant behavior. A sound absorption coefficient of 0.31 further confirms its remarkable acoustic energy dissipation capacity. By leveraging structural design and compositional synergy, this study achieves effective thermal shielding and efficient dissipation of EMW and acoustic energy within a lightweight framework. It presents a new approach for the development of integrated multifunctional aerogel materials and holds great promise for advanced applications in electromagnetic stealth, thermal protection, smart structures, and sustainable protective materials.

## Results and Discussion

### Synthesis and characterization

**Fig. 1.**
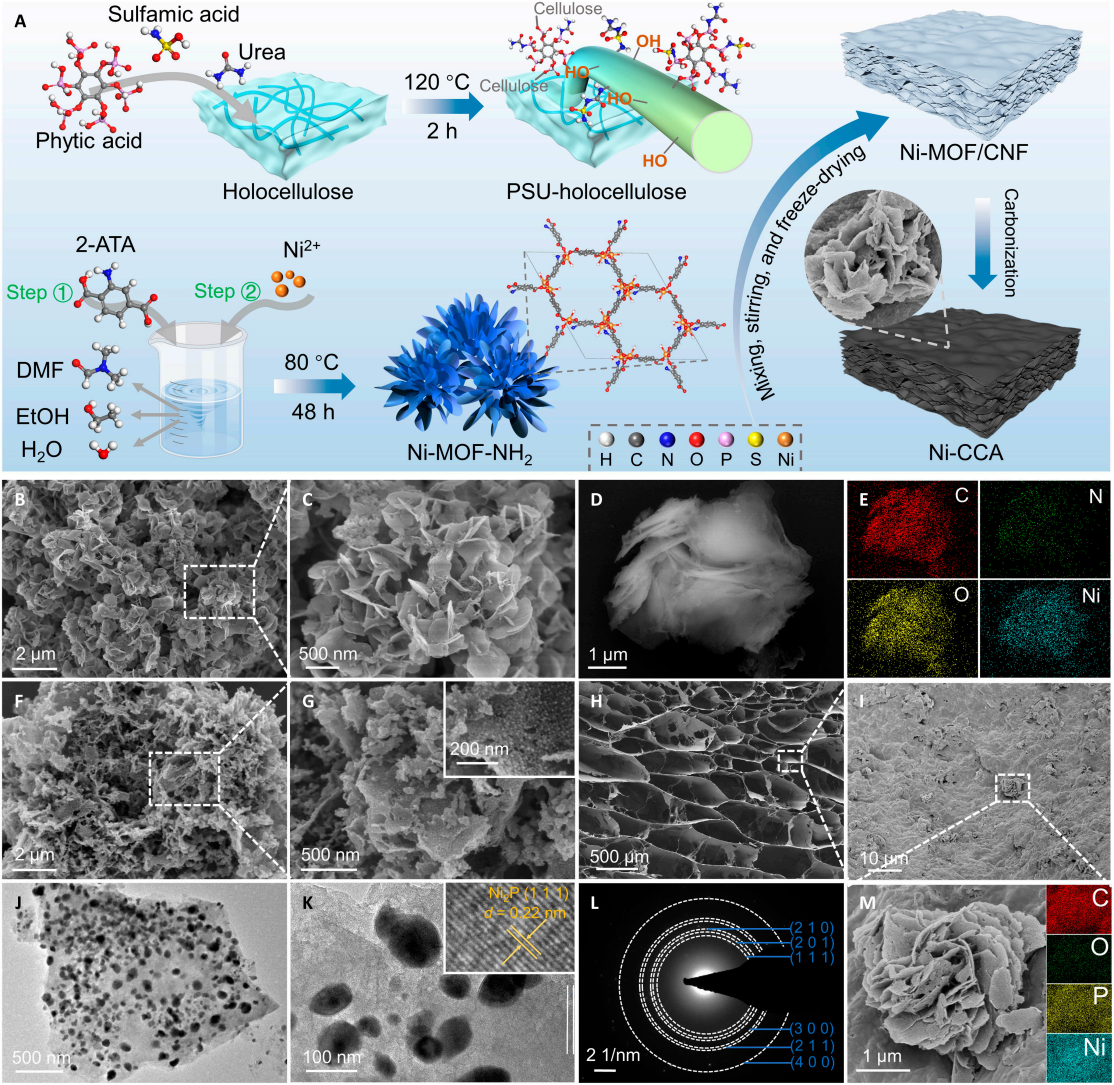
Schematic diagram of the synthesis process of Ni-CCA (A). SEM and EDX images of Ni-MOF-NH_2_ (B to E), Ni-C (F and G), and Ni-CCA (H, I, and M). TEM and diffraction rings of Ni-CCA (J to L).

The synthesis procedure of Ni-CCA is illustrated in Fig. 1A, with detailed protocols provided in the Supplementary Materials. The key steps are as follows: firstly, holocellulose was pretreated with a ternary deep eutectic solvent system composed of P, N, and S, resulting in PSU-holocellulose. This pretreatment not only effectively preserved the original cellulose skeleton but also introduced a variety of polar functional groups (-NH_2_, -SO_3_H, and -PO_4_^3−^), thereby providing a chemical foundation for subsequent interfacial coupling and conductive network formation. Next, 2-ATA and Ni(NO_3_)_2_·6H_2_O were used in a mixed solvent system to synthesize scale-shaped Ni-MOF-NH_2_ via coordination self-assembly. These scale-like flakes were uniformly dispersed in the PSU-holocellulose solution and cross-linked with glutaraldehyde, followed by freeze-drying to obtain the Ni-MOF/CNF composite gel. Finally, a 2-step carbonization strategy was employed to transform Ni-MOF/CNF into Ni-CCA, achieving structural conversion and functional integration.

As shown in Fig. 1C and D and Fig. S2A, the Ni-MOF-NH_2_ precursor exhibits a unique topological structure with scale-like flakes of approximately 200 to 500 nm in size and 20 to 30 nm in thickness. This uniform and loose nanostructure provides an ideal template and metal source for subsequent aerogel formation and carbonization. In Fig. 1F and G, the carbonized Ni-C sample retains the scale features of the MOFs precursor, indicating good structural stability. The final Ni-CCA product (Fig. 1H and I) presents a characteristic 3D layered architecture with robust structural integrity and a multilayer porous network. This honeycomb- and channel-like aerogel framework facilitates multiple reflections of EMW and enhances energy dissipation [28]. Additionally, EDX mapping (Fig. 1E and M) confirms the uniform distribution of C, O, N, and Ni elements, indicating effective nanoscale synergy between the MOF-derived components and the PSU-modified cellulose, forming a functional composite system rich in defects and multiphase interfaces. Notably, high-resolution TEM and selected area electron diffraction (SAED) analyses reveal the crystalline features of Ni-CCA. As shown in Fig. 1J and Fig. S2B and C, the metal nanoparticles are highly dispersed within the carbon matrix, with an average particle size of several tens of nanometers, effectively avoiding agglomeration. The TEM images (Fig. 1K) clearly display lattice spacings of 0.22 nm, corresponding to the (1 1 1) plane of Ni_2_P, confirming the successful transformation of the Ni-MOF-NH_2_ precursor into nickel phosphide nanoparticles during the carbonization process. Furthermore, the inverse fast Fourier transform images and intensity profile analysis in Fig. S3 precisely identify the 0.22-nm lattice fringes, and the ring-like diffraction pattern observed in the SAED (Fig. 1L) further confirms the highly crystalline nature of the resulting Ni_2_P structure.

**Fig. 2.**
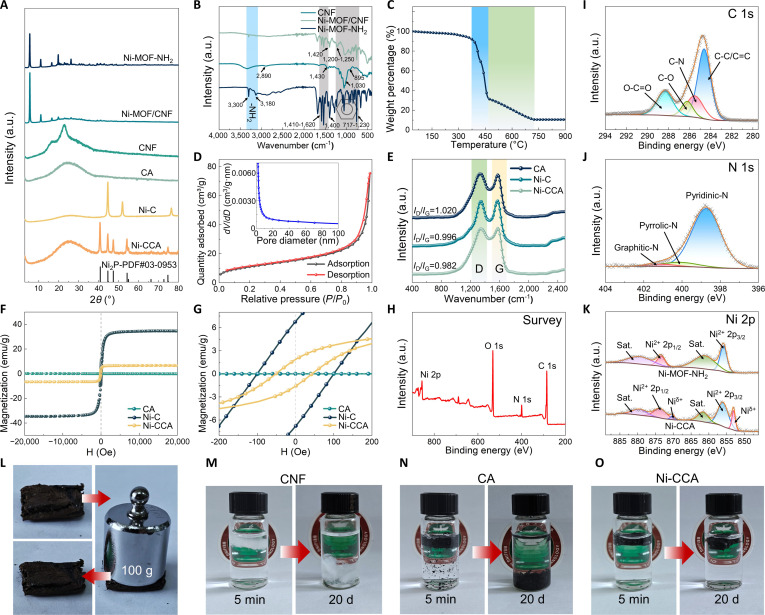
XRD spectra (A), FTIR spectra (B), TGA (C), and BET (D) of Ni-MOF-NH_2_. Raman spectra (E) and VSM (F and G) of CA, Ni-C, and Ni-CCA. XPS spectra of Ni-MOF-NH_2_: survey scan (H), C 1s (I), N 1s (J), and Ni 2p (K). Photos of compression change of Ni-CCA (L). Decomposition of CNF (M), CA (N), and Ni-CCA (O) in water.

The XRD patterns in Fig. 2A systematically reveal the crystalline phase evolution from the MOF precursor to the final carbonized product. Ni-MOF-NH_2_ exhibits distinct diffraction peaks at 2*θ* = 6.5° and 2*θ* = 11.9°, corresponding to the (2 1 0) and (3 0 0) planes of a typical 2D layered MOFs structure [29]. The CNF sample shows a dominant peak at 2*θ* = 22.6°, which is attributed to the (2 0 0) plane of cellulose I-β, while the broad peak near 2*θ* = 16.5° corresponds to the (1 −1 0) and (1 1 0) planes [30]. In the Ni-MOF/CNF composite, the presence of sharp peaks at 6.5° and 11.9°, along with the prominent peak at 2*θ* = 22.6°, indicates the successful integration of MOF crystals onto the cellulose matrix. After carbonization, the CA sample exhibits a broad hump around 2*θ* = 26°, reflecting the transformation of crystalline cellulose domains into an amorphous, heteroatom-doped carbon framework under high-temperature conditions. In the Ni-C pattern, the characteristic MOF peaks disappear completely, and instead, sharp diffraction peaks emerge at 2*θ* = 44.7° (1 1 1), 51.9° (2 0 0), and 76.4° (2 2 0), confirming the reduction of Ni^2+^ ions to face-centered cubic (fcc) metallic Ni (PDF#04-0850)[31]. The Ni-CCA sample shows a broad peak near 26°, attributed to the (0 0 2) plane of graphitized carbon [32]. This peak is slightly narrower than that of CA, suggesting that the MOF-derived carbon framework enhances the graphitic microcrystalline order. Additionally, several new diffraction peaks appear at 2*θ* = 40.7°, 44.6°, 47.3°, 54.2°, 55.1°, and 77.72°, which match well with the standard diffraction data for Ni_2_P (PDF#03-0953) and are consistent with the SAED results [33]. This phase formation is attributed to the solid-state phosphidation reaction between Ni and phosphorus species during the carbonization process, with phosphorus supplied by the PSU-holocellulose matrix.

The Fourier-transform infrared (FTIR) analysis was employed to investigate the interaction mechanism between Ni-MOF-NH_2_ and the CNF, as shown in Fig. 2B. In the CNF sample, absorption peaks at 2,890, 1,430, 1,030, and 895 cm^−1^ are characteristic of cellulose, corresponding to C-H stretching, -CH_2_ bending, C-O-C stretching, and β-glycosidic bond vibrations, respectively [34]. A broad peak around 3,350 cm^−1^ is attributed to the stretching vibration of -OH groups, indicating that the CNF retains a largely intact hydroxyl structure. In contrast, the Ni-MOF-NH_2_ sample exhibits sharp peaks near 3,300 and 3,180 cm^−1^, corresponding to the stretching vibrations of -NH_2_ groups, indicating an abundance of surface amino functionalities [35]. Additionally, peaks at 1,620, 1,540, 1,500, 1,440, and 1,410 cm^−1^ are assigned to aromatic skeletal vibrations, while peaks at 1,230, 1,090, 1,060, 900, 845, 806, 762, and 717 cm^−1^ arise from out-of-plane C-H vibrations of the benzene ring, confirming the presence of organic ligands in the MOFs structure [36]. Vibrational bands near 1,590 and 1,400 cm^−1^ correspond to the asymmetric and symmetric stretching of O-C=O groups, respectively. For the Ni-MOF/CNF composite, the intensity of the original -NH_2_ doublet is markedly reduced and the overall absorption bands broaden and shift toward lower wavenumbers. Simultaneously, new peaks or enhanced peak features appear near 1,200 to 1,250 cm^−1^ and around 1,030 cm^−1^, which can be attributed to the stretching vibrations of P–O–C and S–O–C bonds, respectively. These spectral changes suggest that the -NH_2_ groups in Ni-MOF-NH_2_ likely react with -PO_4_^3−^ and -SO_3_H groups on the PSU-holocellulose surface, forming stable hydrogen-bonded or covalent composite structures. This interaction strengthens the binding between the 2 components, facilitating the stable deposition and integration of Ni-MOF-NH_2_ on the cellulose substrate.

The thermogravimetric analysis (TGA) curves in Fig. 2C systematically reveal the thermal decomposition behavior and component evolution of the Ni-MOF-NH_2_ precursor. From room temperature to approximately 300 °C, the sample shows negligible mass loss, indicating good structural stability of the coordination framework under ambient conditions. A substantial weight loss occurs between 350 and 450 °C (blue-shaded region), corresponding to the decomposition of the organic ligand (2-ATA) and the volatilization of some nitrogen-containing functional groups. Coordinated water and residual solvents are also released in this temperature range. This stage marks the onset of framework collapse and pore structure degradation, indicating that a controlled carbonization process within this window under an inert atmosphere is essential to prevent the migration and aggregation of metal nanoparticles. A second, slower weight loss occurs between approximately 450 and 720 °C (green-shaded region), mainly attributed to further dehydrogenation of the residual carbon skeleton and carbonization of organic fragments. Concurrently, the Ni species gradually transition from oxidized or hydroxide forms to metallic Ni. This stage is crucial for constructing a conductive composite phase and enhancing dielectric and magnetic loss mechanisms.

The specific surface area of Ni-MOF-NH_2_ reaches 392.3 m^2^/g. As shown in the nitrogen adsorption–desorption isotherm (Fig. 2D), a pronounced initial uptake at low relative pressure (*P*/*P*_0_ < ~0.1) indicates the presence of abundant intrinsic micropores within the MOF skeleton. As *P*/*P*_0_ increases to the range of 0.1 to 0.8, the adsorption amount gradually increases, followed by a sharp rise in the high-pressure region (*P*/*P*_0_ > 0.9), accompanied by a slight hysteresis loop. This phenomenon can be attributed to capillary condensation occurring in the mesopores and secondary cavities formed between the self-assembled scale-like flakes. The pore size distribution (inset) shows strong intensity at the small pore size (<5 nm), corresponding to the intrinsic pores of the MOF framework. Signals rapidly decay and tail off toward tens of nanometers, indicating the presence of secondary mesopores and small macropores caused by particle stacking, flake interweaving, and structural defects or cracks. This “intrinsic micropore/structural mesopore” hierarchical pore channel not only ensures a large specific surface area and active site exposure, but also provides a mass transfer channel for subsequent infiltration into the cellulose matrix, element exchange, and carbonized gas escape, which is conducive to maintaining uniform dispersion and slowing down particle agglomeration during the compounding/carbonization process.

To evaluate the structural ordering and defect density of the carbon skeleton, Raman spectroscopy was conducted on CA, Ni-C, and Ni-CCA samples (Fig. 2E). All 3 samples exhibit characteristic D (~1,350 cm^−1^) and G (~1,590 cm^−1^) bands, corresponding to defects and graphitization in carbon materials, respectively [37]. The intensity ratio of the D to G bands (*I*_D_/*I*_G_) is commonly used to estimate defect density. The CA sample shows an *I*_D_/*I*_G_ of 1.020, indicating a relatively high defect level. In contrast, Ni-C and Ni-CCA exhibit lower *I*_D_/*I*_G_ values of 0.996 and 0.982, respectively, suggesting that the catalytic effect of Ni promotes graphitization during carbonization while passivating some structural defects. Notably, the incorporation of heteroatoms such as P, N, and S in Ni-CCA introduces functional polarization sites without markedly disrupting the overall carbon structure. This structural integrity favors the efficient transmission and dissipation of EMW within the material.

The vibrating sample magnetometer (VSM) analysis was conducted to assess the magnetic response behavior of the samples, as shown in Fig. 2F. The CA sample exhibits negligible magnetization, indicating a lack of magnetic properties. In contrast, both Ni-C and Ni-CCA display clear S-shaped magnetic hysteresis loops, confirming their ferromagnetic behavior. Ni-C achieves the highest saturation magnetization (*M*_s_) of 34.6 emu/g, attributed to the presence of a larger quantity of metallic Ni nanoparticles. Although Ni-CCA shows a lower *M*_s_ value of 6.6 emu/g, it still maintains good magnetic responsiveness, mainly due to the partial transformation of Ni into Ni_2_P while retaining a portion of the magnetic phase. Furthermore, as shown in Fig. 2G, the coercivity (*H*_c_) of Ni-CCA is lower than that of CA, indicating enhanced soft magnetic characteristics. This property facilitates its response to alternating electromagnetic fields, thereby promoting magnetic loss mechanisms and enhancing energy dissipation.

To further elucidate the elemental composition and chemical state evolution within Ni-CCA, XPS was performed on both Ni-MOF-NH_2_ and Ni-CCA samples. As shown in Fig. 2H and Fig. S5A, the survey spectra clearly reveal the presence of C 1s, O 1s, N 1s, and Ni 2p signals in both samples. Notably, additional S 2p and P 2p peaks are detected in Ni-CCA, confirming the successful heteroatom doping enabled by deep eutectic solvent pretreatment. High-resolution C 1s spectra (Fig. 2I and Fig. S5B) for both samples display peaks corresponding to C-C/C=C (284.6 eV), C-N (285.6 eV), C-O (286.2 eV), and O-C=O (288.3 eV), indicating that portions of the organic ligand framework are retained post-carbonization [38]. The enhanced intensity of C-N and C-O peaks in Ni-CCA suggests a greater presence of polar functional groups on the carbon skeleton, which is favorable for boosting interfacial polarization loss. The N 1s spectra (Fig. 2J and Fig. S5C) provide insights into nitrogen bonding configurations. In Ni-MOF-NH_2_, nitrogen exists predominantly as pyridinic-N (398.7 eV), pyrrolic-N (400.1 eV), and graphitic-N (401.0 eV) [39]. In Ni-CCA, the distribution shifts notably, with an increased proportion of pyridinic N, which is known for its superior electronic conductivity and polarization-enhancing effects—beneficial traits for enhancing dielectric response performance. The Ni 2p spectra (Fig. 2K) compare the Ni chemical states before and after carbonization. Ni-MOF-NH_2_ exhibits characteristic Ni^2+^ 2p_3/2_ and 2p_1/2_ peaks at 855.9 and 873.5 eV, respectively, along with associated satellite peaks [40]. In Ni-CCA, while some Ni^2+^ signals persist, new peaks at 853.1 and 870.2 eV emerge, attributed to Ni^δ+^ species—indicative of Ni_2_P formation [9]. This observation confirms that Ni^2+^ in the MOF precursor partially transforms into Ni_2_P during thermal treatment. Figure S5D further presents the high-resolution P 2p spectrum of Ni-CCA, where peaks at 129.7 eV (P 2p_3/2_) and 130.5 eV (P 2p_1/2_) correspond to Ni_2_P, while a peak at 133.8 eV denotes P–O bonds, suggesting minor surface oxidation forming phosphate species [41]. These results collectively validate the in situ phosphidation of Ni and the successful incorporation of P and S dopants, which synergistically enhance the functional properties of the composite.

Figure S6 demonstrates the ultralight characteristics of Ni-CCA (*ρ* ≈ 0.0102 g/cm^3^), with a density only 8 times that of air. This feature originates from the 3D porous structure of the MOF-cellulose composite system. The ultralight property does not compromise structural stability, as shown in Fig. 2L, where the material maintains its form even after being compressed under a 100 g load. This can be attributed to the gradient modulus structure formed by glutaraldehyde cross-linking, with MOF scale-like flakes serving as rigid nodes to enhance the local modulus. The water environment decomposition experiment in Fig. 2M to O (from 5 min to 20 days) presented marked differences and the detailed changes can be seen in Fig. S7. CNF absorbs water and swells due to the presence of hydrophilic groups, dissolving on day 3. While CA is delayed in its decomposition due to its carbonization-induced hydrophobicity, the floating debris suggests structural brittleness. In contrast, the Ni-CCA sample maintains relatively good stability in water, showing no discernible dissolution or morphological change even after 20 days, demonstrating strong hydrolytic resistance and chemical stability. In summary, Ni-CCA possesses excellent lightweight properties, compressive strength, and chemical stability, making it highly versatile for a wide range of applications, particularly in areas such as EMW absorption, sensor materials, and marine environments.

### Flame-retardant and thermal insulation performance

**Fig. 3.**
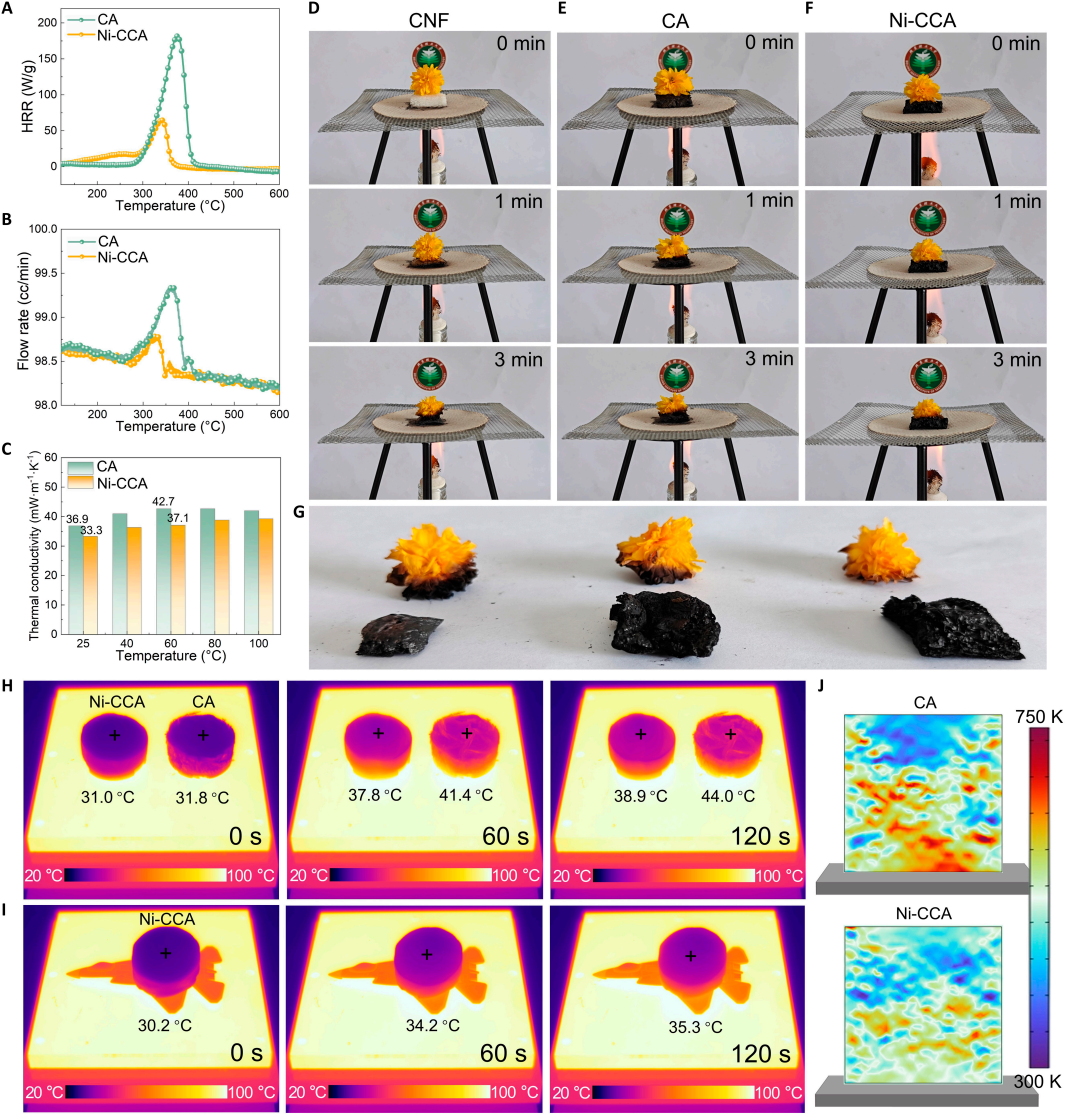
Heat release rate curves (A), flow rate curves (B), and thermal conductivity (C) of CA and Ni-CCA. Digital photos of CNF, CA, and Ni-CCA ignition tests and the appearance of flowers before and after putting them on aerogels (D to G). Infrared thermography (H and I) of samples on the 100 °C heating platform. Simulation of temperature field distribution of CA and Ni-CCA based on COMSOL (J).

Comprehensive data analysis in Fig. 3 demonstrates that the Ni-CCA aerogel exhibits markedly enhanced flame-retardant and thermal insulation properties compared to pristine cellulose-derived materials (CA). The heat release rate (HRR) curves in Fig. 3A reveal that the pHRR of Ni-CCA (64.75 W/g) is markedly lower than that of CA (181.50 W/g), accompanied by a reduction in total heat flux (Fig. 3B). This indicates that the incorporation of MOF-derived scale-like flakes effectively suppresses heat release during combustion. This enhanced flame retardancy is directly attributed to the scale-like carbon barrier derived from the unique hierarchical topology of Ni-MOF-NH_2_ (Fig. 1B and C), which forms during the carbonization process and effectively blocks the release of heat and combustible gases, thereby increasing the carbon residue rate and delaying the spread of flame.

Digital photographs from the ignition test (Fig. 3D to G) further support this conclusion. Under flame exposure, Ni-CCA exhibits only slight surface charring and retains its structural integrity, while CNF and CA undergo rapid ablation and even complete carbonization. This suggests that the flame-retardant property of Ni-CCA stems from its structural stability and thermal barrier effect [36]. Moreover, the yellow flowers on CNF and CA show various degrees of carbonization, whereas those on Ni-CCA remain intact even after 3 min of heating, indicating its effective thermal insulation performance.

The thermal conductivity test (Fig. 3C) and infrared thermal imaging further reveal the low thermal conductivity characteristics of Ni-CCA. Compared to the CA sample, Ni-CCA shows distinct thermal insulation advantages in the temperature range from room temperature to 100 °C. At room temperature, the thermal conductivity of Ni-CCA is 33.3 mW/(m·K), which is lower than CA’s 36.9 mW/(m·K). This difference is primarily attributed to the unique porous structure of Ni-CCA, where the interface scattering effect of the nanoparticles plays an important role in restricting heat transfer, thereby markedly enhancing its thermal insulation performance. Further analysis of the heat transfer process, through comparison of the temperature distribution on the heating platform (Fig. 3H), shows that the surface temperature rise of Ni-CCA is considerably slower. Specifically, at 120 s, the surface temperature of Ni-CCA is 38.9 °C, which is notably lower than CA’s 44.0 °C, indicating that Ni-CCA has superior thermal insulation ability. The heating curves in Fig. S8 intuitively demonstrate that the temperature of Ni-CCA is consistently lower than that of CA, highlighting its sustained thermal management advantage.

The heat transfer behavior of CA and Ni-CCA on a heating stage was further investigated through simulations using COMSOL Multiphysics, with detailed parameter settings provided in the Supplementary Materials. As shown in Fig. 3J, under steady-state heat flux, the CA sample exhibits rapid heat diffusion along the conduction path, resulting in large regions of elevated temperatures. These areas appear orange to yellow in the thermal map, approaching the maximum simulated temperature (750 K), indicating poor thermal shielding performance. In contrast, the temperature distribution in the Ni-CCA sample is substantially lower, predominantly represented by blue to light green regions. This suggests that heat diffusion within the sample is restricted and the thermal conduction process is notably slowed, thereby effectively suppressing temperature rise. The pronounced difference in temperature gradients clearly demonstrates that Ni-CCA exhibits superior thermal insulation performance under the same heat flux conditions.

Moreover, Fig. 3I demonstrates the application of Ni-CCA on a fighter jet model. When comparing the areas covered by Ni-CCA with the exposed areas, the temperature increase in the Ni-CCA-covered areas is notably lower than in the exposed regions, with the color matching the low-temperature background, indicating excellent infrared stealth performance. This result further validates the thermal shielding capability of Ni-CCA, emphasizing its potential in thermal protection and stealth technology [42].

### Sound absorption performance

**Fig. 4.**
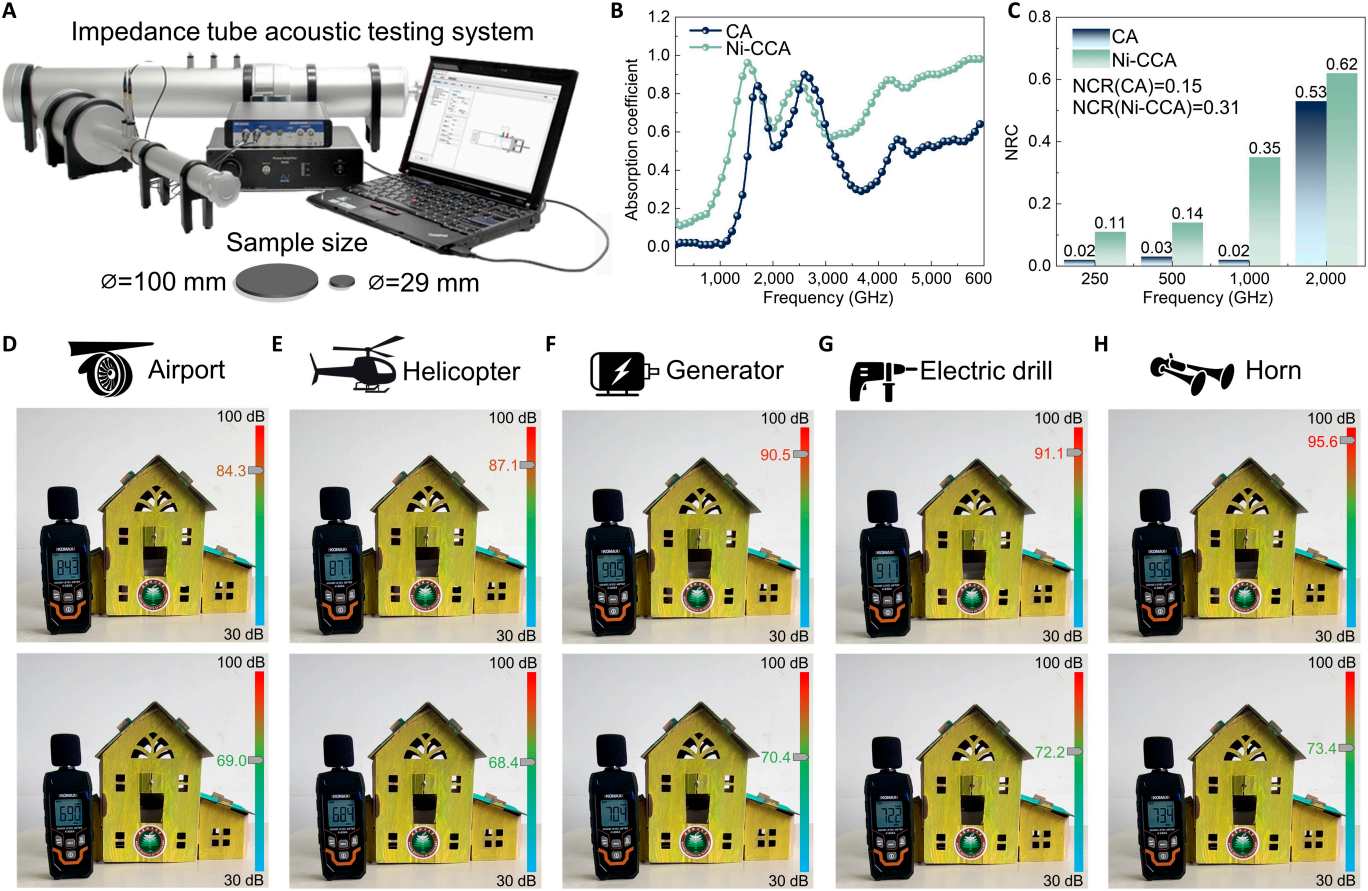
Impedance tube acoustic test system (A), and sound absorption coefficient curves (B) and NRC (C) of CA and Ni-CCA. Noise reduction test of Ni-CCA in different scenes: airport (D), helicopter take-off (E), generator (F), electric drill (G), and truck horn (H).

Figure 4 illustrates the acoustic performance of CA and Ni-CCA. An impedance tube testing system (Fig. 4A) was employed to measure the sound absorption coefficients of the samples across various frequencies, using circular samples with diameters of 100 and 29 mm for low- and high-frequency tests, respectively. As shown in Fig. 4B, Ni-CCA exhibits considerably higher sound absorption coefficients than CA over a broad frequency range (below 1,500 Hz and above 3,000 Hz). While CA reaches a peak absorption coefficient of approximately 0.9 (2,663 Hz), Ni-CCA approaches 1.0 (1,510 Hz), indicating near-total sound absorption. This improvement is primarily attributed to the introduction of Ni-MOF-NH_2_ and the formation of a hierarchically porous network structure (Fig. 1F and G) during carbonization, which greatly enhances acoustic impedance matching and internal friction, thereby facilitating effective sound energy attenuation. Furthermore, the noise reduction coefficient (NRC), an index used to evaluate the acoustic performance of porous materials, is presented in Fig. 4C. NRC is calculated as the average of the material’s absorption coefficients at 250, 500, 1,000, and 2,000 Hz [43]. Ni-CCA achieves an NRC of 0.31 (more than twice that of CA [0.15]), indicating a substantial enhancement in sound absorption across a wide frequency spectrum. Notably, its NRC at high frequency (2,000 Hz) reaches 0.62, highlighting Ni-CCA’s considerable potential for noise control applications in architectural acoustics and transportation environments.

To further validate the material’s practical noise reduction capability, Ni-CCA was applied in simulated scenarios representing different noise sources (Fig. 4D to H). In environments with intense noise such as airports, helicopters, generators, electric drills, and truck horns, the noise levels without coverage ranged between 84 and 96 dB. After applying Ni-CCA, the noise levels dropped substantially to 69.0, 68.4, 70.4, 72.2, and 73.4 dB, respectively. These reductions, spanning 15 to 23 dB, clearly demonstrate the material’s outstanding ability to attenuate sound energy. It is worth noting that Ni-CCA shows greater suppression for high-frequency noise (e.g., electric drills and horns) than for low-frequency sources (e.g., generators), which is consistent with the trend observed in impedance tube tests, where higher absorption was achieved in the high-frequency range.

### Electromagnetic performance and RCS simulation results

**Fig. 5.**
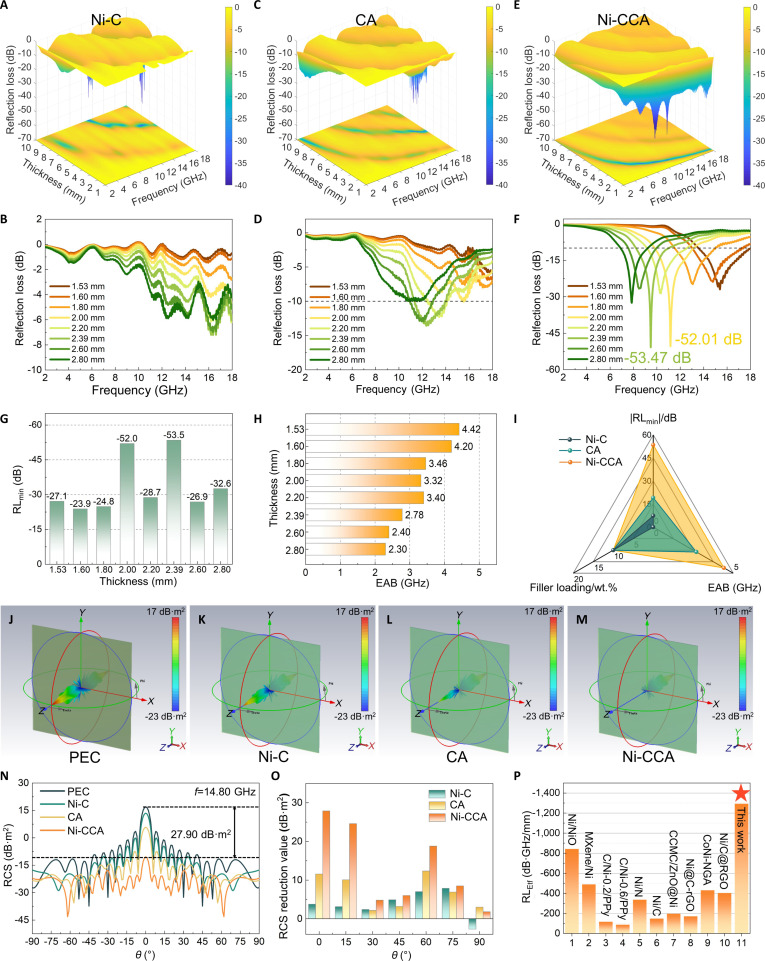
RL plots of 5 wt.% loading Ni-C, CA, and Ni-CCA (A to F). RL_min_ diagram (G) and EAB diagram (H) of Ni-CCA at different thicknesses. EMW performance radar chart of Ni-C, CA, and Ni-CCA (I). RCS simulation of PEC, Ni-C, CA, and Ni-CCA (J to M) and RCS curve (N). RCS reduction value of Ni-C, CA, and Ni-CCA compared with PEC (O). RL_Etf_ comparison diagram (P) of previous similar aerogels.

RL is a key parameter for evaluating the EMW absorption performance of materials [44], with the relevant electromagnetic performance calculation formulas provided in the Supplementary Materials. The 3D RL maps (Fig. 5A to F) demonstrate that at a 5 wt.% filler loading, Ni-CCA exhibits excellent broadband absorption performance within the 2 to 18 GHz frequency range. It achieves a minimum RL (RL_min_) value of −53.47 dB at a thickness of 2.39 mm, markedly outperforming Ni-C (−7.24 dB) and CA (−13.63 dB). The 2D RL curves (Fig. S9) further reveal that Ni-CCA maintains RL values below −20 dB (corresponding to 99% EMW absorption) across a thickness range of 1.5 to 4.0 mm. As shown in Fig. 5G and H, Ni-CCA achieves 2 prominent RL_min_ peaks at thicknesses of 2.0 and 2.39 mm, reaching −52.02 dB and −53.47 dB, respectively. The corresponding EAB (RL < −10 dB) reaches up to 4.42 GHz.

The radar chart in Fig. 5I compares the EMW absorption performance of Ni-C, CA, and Ni-CCA, clearly showing Ni-CCA’s absolute advantages in both RL_min_ and EAB. These enhancements are attributed to the 3D cross-linked network structure and the multi-scattering-absorption paths facilitated by the scale-like flakes. Notably, at a low filler loading of only 5 wt.%, Ni-CCA achieves an RL_min_ of −53.47 dB, which is comparable to or even exceeds that of most reported aerogel-based absorbers requiring much higher loadings (typically 15 to 45 wt.%), corresponding to an approximately 2- to 15-fold improvement in material efficiency. To comprehensively evaluate Ni-CCA’s overall EMW absorption performance, it was further compared with representative aerogel-based materials reported in recent literature. The comparative data are listed in Table S1 and visually summarized in Fig. S10. In terms of key absorption indicators, Ni-CCA achieves an RL_min_ of −53.48 dB, outperforming most comparable materials and second only to systems with substantially higher filler contents—demonstrating its strong absorption capability even at a low loading (5 wt.%).

Regarding bandwidth, Ni-CCA reaches an EAB of 4.42 GHz, on par with most of the referenced materials. Additionally, its corresponding thickness (*T*_EAB_ = 1.53 mm) is relatively thin, highlighting its potential for use in lightweight and compact EMW absorbers. Figure S10A evaluates the overall performance of various materials across 5 key metrics: |RL_min_|, RL_min_ corresponding thickness (*T*_RLmin_), EAB, *T*_EAB_, and filler loading. Ni-CCA (green area) shows balanced optimization across multiple dimensions, with notable advantages in absorption intensity, bandwidth, and low filler content. Figure S10B further compares RL and EAB values, confirming that Ni-CCA achieves strong absorption while maintaining a broad bandwidth. To assess performance under normalized conditions, a new metric RL_Etf_ [RL_Etf_ = (RL_min_ × EAB)/(*T*_RL_ × *T*_EAB_ × filler loading)] was introduced. At 5 wt.% loading, Ni-CCA shows a notably higher RL_Etf_ than other reported aerogel systems (Fig. 5P), indicating its great potential as a lightweight and efficient EMW absorbing material.

Furthermore, CST Studio Suite was used to simulate the radar cross-section (RCS) of typical structures (Fig. 5J to M). At 14.80 GHz, Ni-CCA exhibits the weakest radiation lobe, indicating that its absorption layer effectively reduces incident wave reflection and backscattering compared to perfect electric conductor (PEC), Ni-C, and CA—demonstrating its electromagnetic stealth capability. The RCS distribution curves (Fig. 5N) and RCS reduction value bar chart (Fig. 5O) clearly show that Ni-CCA effectively suppresses RCS compared to PEC across incident angles from −90° to 90°, with a maximum suppression of up to 27.90 dB·m^2^, thus substantially reducing radar detectability.

**Fig. 6.**
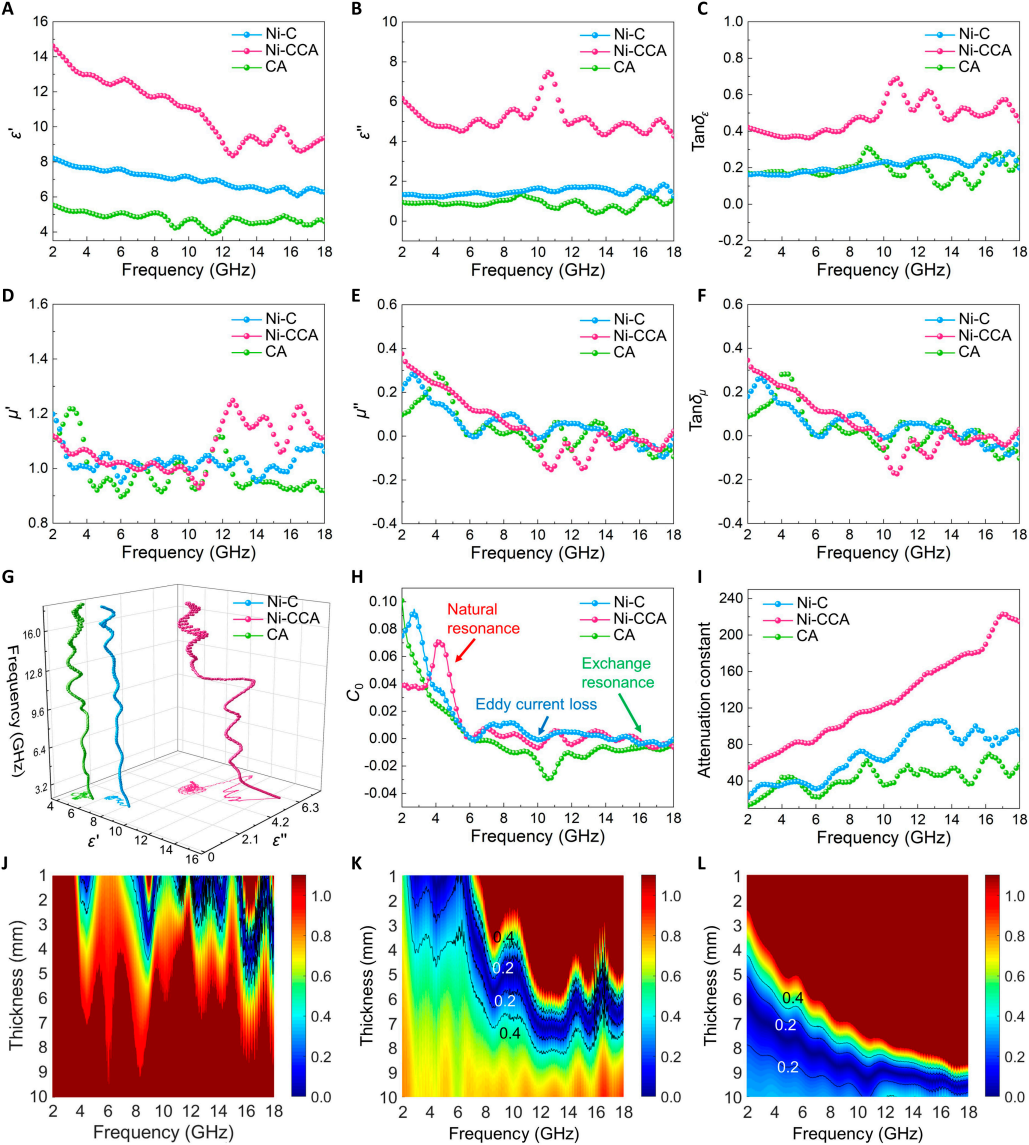
Loading (5 wt.%) of Ni-C, CA, and Ni-CCA: *ε*′ (A), *ε*″ (B), Tan*δ_ε_* (C), *μ*′ (D), *μ*″ (E), and Tan*δ_μ_* (F). 3D Cole–Cole curve (G), *C*_0_ (H), attenuation constants (I), and |Δ| diagrams (J to L).

To reveal the origin of the excellent EMW absorption performance of Ni-CCA, electromagnetic parameter analysis was conducted as shown in Fig. 6. The primary mechanisms of EMW absorption are dielectric loss and magnetic loss, and their deep coupling collectively contributes to strong EMW absorption performance [45]. In terms of dielectric properties (Fig. 6A and B), Ni-CCA exhibits consistently and considerably higher *ε*′ and *ε*″ across the entire 2 to 18 GHz range compared to Ni-C and CA, with a mild decrease and several oscillation peaks as the frequency increases. On one hand, this indicates that Ni-CCA has a stronger ability to store electromagnetic energy (higher *ε*′), while on the other hand, it demonstrates that its loss component (*ε*″) is sufficient for effective electromagnetic dissipation. The peak–valley oscillations typically result from the coupling of various relaxation processes (dipolar orientation and interfacial polarization) with conductive losses. This is because the deep eutectic solvent pretreatment introduces polar functional groups such as P/N/S into the cellulose framework, and after carbonization, defect-doped carbon frameworks are formed. Additionally, the Ni-MOF-NH_2_ scale-like flakes and PSU-cellulose/carbon sources synergistically undergo carbonization to form multilevel hybrid interfaces (Ni_2_P/C, heteroatom-doped regions/amorphous carbon regions), which generate Maxwell–Wagner–Sillars polarization under alternating electric fields [46]. Meanwhile, defects and local dipoles formed by the carbonization of residual O/P-coordinated and amino groups also contribute to oriented polarization [47]. In contrast, CA only possesses a PSU-cellulose-derived carbon network with a limited number of polarization centers, resulting in moderate *ε*′ and *ε*″ values; Ni-C primarily originates from the Ni-MOF-derived carbon shell and Ni particles, lacking a continuous polar polymer–inorganic interface network, thus exhibiting the lowest dielectric constants. In Fig. 6C, Tan*δ_ε_* results further confirm these differences, with Ni-CCA maintaining a higher Tan*δ_ε_* value accompanied by several localized peaks, reflecting strong and diverse dielectric loss channels. Ni-C and CA are limited by the lack of polarization centers or continuity of conductive networks, resulting in weakened loss capabilities.

In terms of magnetic properties, the *μ*′ values of all 3 samples are close to 1 (Fig. 6D), indicating that the overall system exhibits weak magnetic/paramagnetic responses. However, *μ*″ (Fig. 6E) shows fluctuations in certain frequency bands, especially for Ni-C and Ni-CCA, indicating that residual Ni and its oxidation/carbonization products still generate some magnetic loss. In Ni-CCA, Ni_2_P is dispersed and anchored within the porous carbon/heteroatom-doped carbon matrix (as confirmed by TEM in Fig. 1J and K), with a wider particle size distribution and more complex interface coupling, triggering natural resonance (low-frequency region) and exchange resonance/coercivity-related magnetic losses (higher-frequency region) over a broader frequency range. Although Tan*δ_μ_* is generally lower than Tan*δ_ε_*, in EMW absorption composites, moderate magnetic loss can synergize with dielectric loss, preventing the impedance mismatch commonly found in purely dielectric absorbers.

The 3D Cole–Cole plot (Fig. 6G) roughly compares the relaxation arc trends. The Ni-CCA curve presents continuous multistage arc segments with a local deviation from the ideal arc, indicating the simultaneous presence of multiple polarization/relaxation processes in the system. The 2D Cole–Cole plot provides an intuitive comparison of the number of relaxation arcs. An ideal single Debye relaxation corresponds to a complete semicircle, while multiple relaxations appear as a staircase pattern or a series of semicircles connected in tandem [48]. All the curves in Fig. S11 show characteristics of “multiple relaxation series” and “conductive tailing”, indicating that the dielectric loss of the samples results from the synergistic effect of polarization relaxation loss and conduction loss [49]. In Fig. S11A, it can be seen that Ni-C exhibits several small semicircles in the *ε*′ ≈ 4 to 5 region, with limited amplitude, indicating a small number of polarization centers that can be excited in the system and weaker relaxation intensity. In Fig. S11B, the number and scale of the semicircles for CA are larger compared to Ni-C (*ε*′ ≈ 6 to 7), indicating that the PSU-modified cellulose leaves a large number of heteroatoms, defects, and pore/wall interfaces after carbonization, which can trigger stronger interfacial polarization and dipole orientation relaxation. The Cole–Cole curve for Ni-CCA (Fig. S11C) is the most complex, with dense and large amplitude arc segments gathered in the *ε*′ ≈ 9 to 10 region, indicating the coexistence of multilevel polarization processes. These heterogeneous interfaces and defect dipoles repeatedly polarize-relax under the alternating field, providing sufficient channels for broad-frequency dielectric loss. More notably, the curve exhibits a long-range conductive tail in the high *ε*′ side (~10 to 14), indicating the gradual activation of charge migration/jump conduction pathways as the frequency changes. Ni catalyzes localized graphitization during the carbonization process, improving the connectivity of the carbon network and markedly enhancing the contribution of conduction loss.

The *C*_0_ curve (Fig. 6H) provides clues to distinguish eddy current loss from resonance-type magnetic loss [50]. If pure eddy current loss dominates, *C*_0_ should remain approximately constant across a wide frequency range. In reality, the Ni-CCA curve shows obvious fluctuations with frequency, along with several peaks. The fluctuations in the lower- and higher-frequency regions reflect multilevel resonance caused by differences in the size, crystal anisotropy and magnetic exchange coupling of Ni_2_P nanoparticles. The intermediate frequency range still retains a certain slope, indicating that eddy currents also contribute in the conductive carbon framework and metal/carbon interfaces. Ni-C also shows some resonance peaks, but due to the lack of a hierarchical conductive network and heterogeneous interface regulation, the peaks are narrower and sharper. CA, on the other hand, almost exhibits a low-amplitude baseline, consistent with its nonmagnetic carbon source characteristics.

The attenuation constant (*α*) and |Δ| represent the inherent ability of a material to dissipate electromagnetic energy and the degree of impedance matching between incident-reflected EMW at the interface, respectively [51]. A |Δ| value close to zero indicates that most incident EMW enter the material rather than reflecting off its surface, which is crucial for efficient absorption. Simply pursuing a high *α* value may otherwise lead to severe impedance mismatch, resulting in strong surface reflection and reduced absorption efficiency. Figure 6I shows that the *α* value of Ni-CCA gradually increases with frequency, consistently higher than that of Ni-C and CA, indicating that the multiple loss mechanisms and better conductive/polarization synergy within Ni-CCA substantially enhance the EMW attenuation capability. Figure 6J to L display the |Δ| distribution for different thicknesses, where the dark blue area (|Δ| → 0) represents good impedance matching, which facilitates the coupling of the incident wave into the material rather than reflection at the interface. Ni-CCA (Fig. 6L) shows relatively small |Δ| values across a wide thickness window (especially in the 2 to 5 mm range) and broad frequency bands, suggesting that its dielectric/magnetic parameter combination is more balanced. This is related to its “moderately high” but not excessive dielectric constant, moderate magnetic loss, and the effective dielectric adjustment caused by its porous, lightweight framework. CA (Fig. 6K) exhibits a relatively narrower matching range that requires a larger thickness, while Ni-C (Fig. 6J) has a fragmented and narrow matching window. The improvement in impedance matching explains why Ni-CCA still achieves excellent RL performance and a high RL_Etf_ value under low filler (5 wt.%) conditions.

To demonstrate that 5 wt.% is the optimal filler content for this system, the EMW absorption performance of Ni-C, CA, and Ni-CCA with low (1 wt.%) and high (10 wt.%) filler loading was compared. When the filler content was reduced to 1 wt.%, Ni-CCA still exhibited superior EMW-absorbing performance compared to Ni-C and CA (see Fig. S12), but its RL isosurface (RL < −10 dB) became more scattered, appearing as narrow stripes and EAB sharply contracted. This indicates that at lower filler content, it is difficult to form an effective conductive-polarization loss network, limiting the EMW-absorbing performance. RCS simulations (Fig. S13) further show that 1 wt.% Ni-CCA weakened its effect on backscattering lobes, with the maximum suppression value not exceeding 20 dB·m^2^. In Fig. S14A, the attenuation constants of CA and Ni-CCA are similar, but Ni-CCA’s impedance matching |Δ| value shows more ideal performance (Fig. S14B to D); thus, Ni-CCA performs better in EMW absorption than CA at 1 wt.% filler content.

In contrast, when the filler content was increased to 10 wt.% (Fig. S15), CA showed a noticeable RL absorption peak at this concentration, indicating that with an increase in filler content, CA’s EMW-absorbing performance improved to some extent. In comparison, the RL peak and EAB of Ni-CCA further decreased, exhibiting negligible EMW-absorbing performance, primarily due to impedance mismatch effects triggered by the excessively high filler content. RCS simulation data (Fig. S16) show that both Ni-C and Ni-CCA samples had almost no suppression effect on the incident wave, while CA’s maximum suppression value was only 11 dB·m^2^. Combining the attenuation constant and impedance matching analysis (Fig. S17), the attenuation constant of Ni-CCA showed a substantial increase, indicating an enhanced ability of the material to dissipate EMW energy. However, at 10 wt.% filler content, Ni-CCA’s |Δ| value deviates substantially from zero over most of the frequency range, indicating a severe deterioration in impedance matching characteristics. This leads to interface reflection, preventing EMW from entering the material and thereby severely weakening the EMW absorption effect. In conclusion, the 5 wt.% Ni-CCA filler content strikes a better balance between attenuation ability and impedance matching, making it the optimal filler ratio.

The layered micro/nano structure, rich dopant incorporation, and dielectric/magnetic coupling synergy endow Ni-CCA with exceptional EMW absorption, as well as infrared and electromagnetic stealth capabilities. Figure S18 systematically explains the stealth mechanism of Ni-CCA aerogel from a multi-physical-field coupling perspective. In the EMW frequency range (2 to 18 GHz), the material achieves broadband EMW absorption through multiple loss mechanisms: the hierarchical porous structure of the aerogel and MOF-derived scale-like flakes (micro-pores, meso-pores, and macro-pores) forms a refractive index gradient layer, causing the incident EMW to undergo a smooth transition from “free space/macro-pores/meso-pores/micro-pores”. This promotes repeated internal reflection, scattering, and deflection of EMW, thereby extending the propagation path, while the high *α* value leads to deep attenuation.

The high-conductivity carbon matrix derived from the holocellulose pyrolysis provides a continuous network for electron transport. Free electrons undergo migration and collisions under the alternating electromagnetic field, converting electromagnetic energy into thermal energy. The introduction of a large number of defect dipoles (such as pyridine nitrogen, C=O bonds) and heteroatom doping (e.g., P/N/S) through PSU-holocellulose brings in polar groups that easily undergo orientational polarization in the electromagnetic field, thereby enhancing dipole polarization. Interface polarization occurs at the Ni_2_P nanoparticle–carbon matrix heterojunction and the accumulated space charge relaxes and dissipates energy under electromagnetic field oscillation.

Furthermore, the uniformly embedded Ni nanoparticles derived from Ni-MOF-NH_2_ scale-like flakes act as magnetic cores, inducing natural magnetic resonance and eddy current loss, compensating for the lack of magnetic response in pure carbon systems [52]. Notably, the Ni nanoparticles (magnetic loss units) derived from the MOFs and the carbon skeleton (dielectric loss units) form a composite system and the tailored complex dielectric constant (*ε*_r_) and complex permeability (*μ*_r_) achieve excellent impedance matching, allowing more EMW energy to enter the material for dissipation.

Additionally, the innovation of Ni-CCA is also reflected in the cross-scale synergy between infrared camouflage and electromagnetic stealth. The layered porous carbon framework with low thermal conductivity suppresses heat conduction and surface radiation, effectively reducing infrared characteristics under external thermal stimuli. The temperature of the aerogel cladding remains notably lower after the same heating, making it less detectable in infrared thermal imaging. This behavior is crucial for thermal camouflage, especially in military applications that require low infrared emissivity. The outstanding EMW absorption performance of the Ni-CCA cladding, along with its remarkable RCS reduction effect, allows the target to conceal its presence in radar detection images, demonstrating its potential application in electromagnetic stealth technology.

In summary, the superior performance of Ni-CCA originates from the synergistic effect of its structure and composition. The hierarchical scale-like Ni-MOF-NH_2_ not only provide abundant interfaces and active sites for EMW dissipation, but also serve as precursors for magnetic Ni species after carbonization. The covalently cross-linked PSU-holocellulose matrix ensures the mechanical and thermal stability of the aerogel. The hybridization of dielectric carbon and magnetic nanoparticles forms a microcapacitor and microinductor network that enhances attenuation ability across a wide frequency range. This design strategy, featuring multicomponent synergy, hierarchical structural matching, and multidimensional functional coupling, enables Ni-CCA to serve as a multifunctional platform for next-generation stealth materials.

## Conclusion

This study successfully constructs a lightweight, multifunctional aerogel (Ni-CCA) by integrating hierarchical Ni-MOF-NH_2_ scale-like units into a sustainable cellulose matrix. The key innovation lies in the rational design of a hierarchical MOF-cellulose architecture via a scalable assembly–carbonization strategy, which enables the synergistic integration of EMW absorption, flame retardancy, thermal insulation, and acoustic damping within a single, low-density bio-aerogel. Notably, this multifunctionality is achieved at a very low filler loading, highlighting the material’s efficiency and sustainability advantages. Benefiting from hierarchical porosity, abundant heterointerfaces, and synergistic dielectric–magnetic coupling, the resulting aerogel successfully achieves integrated multifunctionality, including effective EMW attenuation, enhanced flame retardancy, low thermal conductivity, and efficient acoustic energy dissipation within a lightweight framework. This multifunctional integration demonstrates that complex, multi-physical-field protection can be realized without relying on high filler contents, addressing a key challenge in the development of next-generation protective materials. This work demonstrates the feasibility of using MOF-biopolymer hybrid aerogels as a versatile platform for next-generation protective materials. The proposed strategy is readily extendable to other MOF chemistries and biomass matrices, offering opportunities for tunable multifunctionality, scalable fabrication, and application-driven optimization. Such materials hold considerable promise for advanced applications in electromagnetic stealth, thermal protection, noise mitigation, and sustainable smart structures operating under complex multi-physical-field environments.

## Materials and Methods

This section is provided in the Supplementary Materials.

## Data Availability

All data are available in the manuscript or the Supplementary Materials or from the authors.

## Supplementary Materials

Supplementary 1 Figs. S1 to S18 Table S1 References [53–61]
