# Supplementary material for: Designing MOF-Cellulose Bio-Aerogels for Electromagnetic Management and Fire-Acoustic Safety
Source: Research (Wash D C). 2026 Feb 6;9:1111. doi: 10.34133/research.1111 (PMC12876565; doi:10.34133/research.1111)
Supplement: Supplementary 1 — Figs. S1 to S18 Table S1 References [53–61] [file research.1111.f1.zip › Supplementary Materials.docx]

Supplementary Materials for

**Designing MOF-Cellulose Bio-Aerogels for Electromagnetic Management and Fire-Acoustic Safety**

**Authors:** Jinhu Hu^1,2,†^, Jierui Ye^1,†^, Pooya Jafari^3^, Boyou Hou^3^, Jinfeng Li^4^, Toan Dihn^3^, Jiao Liu^5^, Pan Chen^1,*^, Mingliang Ma^5^, Min Hong^6^, Ye-Tang Pan^1,3,*^, Pingan Song^3,*^

**Affiliations:**

^1^School of Materials Science & Engineering, Beijing Institute of Technology, Beijing 100081, China

^2^National Engineering Research Center of Flame Retardant Materials, School of Materials Science & Engineering, Beijing Institute of Technology, Beijing 100081, China

^3^Centre for Future Materials, School of Agriculture and Environmental Science, University of Southern Queensland, Springfield, Queensland 4300, Australia

^4^Electronic Information School, Wuhan University, Wuhan 430072, Hubei, China

^5^School of Civil Engineering, Qingdao University of Technology, Qingdao 266520, Shandong, China

^6^Centre for Future Materials, School of Engineering, University of Southern Queensland, Springfield, Queensland 4300, Australia

† The two authors contributed equally to this paper.

^*^Address correspondence to: Pan Chen; 6120190049@bit.edu.cn, Ye-Tang Pan; pyt@bit.edu.cn and Pingan Song; [pingan.song@usq.edu.au](mailto:pingan.song@usq.edu.au), pingansong@gmail.com

**Nomenclature of key samples and materials**

| **Abbreviation** | **Full Name / Description** |
| --- | --- |
| Holocellulose | Raw material containing cellulose (79 wt.%) and hemicellulose (21 wt.%) |
| PSU-holocellulose | Holocellulose pretreated with a ternary deep eutectic solvent (Phytic acid, Sulfamic acid, Urea) |
| Ni-MOF-NH_2_ | Scale-like nickel-based metal–organic framework synthesized from Ni^2+^ and 2-aminoterephthalic acid (2-ATA) |
| CNF | Cellulose nanofibrils |
| Ni-MOF/CNF | Composite precursor aerogel formed by embedding Ni-MOF-NH_2_ into the CNF network |
| CA | Carbon aerogel derived from CNF after two-step carbonization |
| Ni-CCA | Ni-MOF-derived/cellulose-based composite aerogel |
| 2-ATA | 2-aminoterephthalic acid |
| EtOH | Anhydrous ethanol |
| DMF | N,N-dimethylformamide |
| PEC | Perfect electric conductor |

**Supplementary Experimental Sections:**

**1. Raw Materials**

Holocellulose was purchased from Sichuan Yibin Paper Co., Ltd., China. Phytic acid (70% in H_2_O), urea and amidosulfonic acid were purchased from Aladdin Reagents (Shanghai) Co., Ltd.

2-aminoterephthalic acid (2-ATA) was purchased from Shanghai Bide Pharmaceutical Technology Co., Ltd. Nickel nitrate hexahydrate (Ni(NO_3_)_2_·6H_2_O), N,N-dimethylformamide (DMF) and anhydrous ethanol (EtOH) were provided by Beijing Tongguang Fine Chemicals Company. Glutaraldehyde was sourced from Shanghai Merck Chemical Technology Co., Ltd.

**2. Synthesis of PSU-holocellulose**

Following the method described in previous work[34], PSU-holocellulose was prepared. Specifically, phytic acid, amidosulfonic acid and urea were mixed in a molar ratio of 1:1:3 in a beaker and stirred at 80℃ to obtain a transparent solution (PSU solution). Subsequently, holocellulose was added to the PSU solution and reacted at 120℃ for 2 h. After the reaction, deionized water was added to terminate the reaction and the sample was washed repeatedly until the filtrate reached a neutral pH. The resulting solution contained PSU-holocellulose.

**3. Synthesis of Ni-MOF-NH_2_**

In a mixed solvent containing DMF (60 mL), ethanol (5 mL) and deionized water (5 mL), 2-ATA (1.36 g, 0.75 mmol) was added and stirred to form a homogeneous solution. Then, Ni(NO₃)₂·6H₂O (2.18 g, 0.75 mmol) was added and the mixture was stirred for 48 h. The precipitate obtained through centrifugation and filtration was washed three times with ethanol and deionized water. Finally, the sample was dried in a vacuum oven at 60℃ for 12 h to obtain Ni-MOF-NH_2_.

**4. Synthesis of Ni-CCA**

1 g of Ni-MOF-NH_2_ was added to 10 mL of deionized water and stirred for 30 min. The resulting solution was then slowly added to 49 g of the PSU-holocellulose solution. After stirring for 30 min at 40℃, 2 mL of a 5% (v/v) glutaraldehyde solution was added dropwise and the mixture was stirred for an additional hour. The solution was transferred to a -10℃ freezer and frozen for 10 h, followed by freeze-drying to obtain Ni-MOF/CNF. For comparison, the PSU-holocellulose solution was treated in the same manner and the dried sample was named cellulose nanofibrils (CNF). Ni-CCA was obtained by carbonizing Ni-MOF/CNF. The sample was first heated to 280℃ in air at a heating rate of 5℃/min and held for 1 h to stabilize the fiber skeleton structure. It was then heated to 800℃ in a N_2_ atmosphere at a rate of 5℃/min and held for 2 h. Similarly, Ni-MOF-NH_2_ and CNF were carbonized under the same conditions, resulting in samples named Ni-C and CA, respectively.

**5. Materials Characterization**

The morphology and microstructure of all samples were observed using a field-emission transmission electron microscope (TEM, FEI Tencai G2 F20) and a scanning electron microscope (SEM, Hitachi SU8020) equipped with an energy-dispersive X-ray (EDX) detector. X-ray diffraction (XRD) patterns were collected using a Rigaku MiniFlex 600 powder X-ray diffractometer, with a 2θ range of 2-80° and a scan rate of 10°/min. The structural information of the nanofibrils was characterized using nuclear magnetic resonance (NMR, Bruker AVANCE NEO 400 WB) spectroscopy. Fourier-transform infrared (FTIR) spectra were obtained using an NLCOLET 6700 IR spectrometer in the range of 400-4000 cm^−1^. Raman spectra in the range of 400-3000 cm^−1^ were tested using a Renishaw inVia InSpect spectrometer with a 532 nm laser excitation. X-ray photoelectron spectroscopy (XPS) data were characterized on a Thermo ESCALAB 250Xi instrument. Nitrogen adsorption isotherms and pore size distributions were collected using a multi-station fully automatic surface area and porosity analyzer (ASAP 2460 3.01). Thermogravimetric analysis (TGA) data were obtained on a Netzsch 209 F1 thermal analyzer under a nitrogen atmosphere, with a heating rate of 10℃/min from 40℃ to 900℃. The static magnetic properties of the samples were measured using a vibrating sample magnetometer (VSM, SQUID-VSM MPMS-3y). The heat release data were collected using a micro-calorimeter (Govmark MCC-2). The heat transfer process of the samples on a heating platform was recorded using an infrared thermal imaging system (FLIR T530). The thermal conductivity of the samples was measured using a thermal constant analyzer (Hot Disk TPS2200). The sound absorption performance of the materials was evaluated by measuring the sound absorption coefficient using a transfer function impedance tube (BSWA SW4601/SW4661).

**6. COMSOL Multiphysics Simulation Modeling Process Parameters**

In this study, thermal conduction behavior was simulated using COMSOL Multiphysics 6.2 software with the Heat Transfer in Solids module. First, the module was selected and set for steady-state simulation, which is appropriate for investigating the thermal conduction characteristics in this study. Based on the simulation requirements, conduction was chosen as the primary physical field and appropriate treatments were applied to heat flux and convection. In terms of geometric modeling, a simplified 2D square geometry was selected to simulate the thermal conduction process of the material. The thermal physical properties of the material, including thermal conductivity (k), density (ρ) and specific heat capacity (Cp), were input based on experimental results and supplemented with data from the built-in material library of COMSOL. To ensure the accuracy of the simulation, corresponding parameters were also adjusted according to experimental conditions and material properties. In terms of boundary condition setting, Dirichlet conditions were adopted for the temperature boundary and the initial temperature (T = 300K) was set with a simulation time of 30 s. The heat flux boundary used a Neumann condition to simulate heat input (q = 1000 W/m^2^). Additionally, a convective boundary was introduced, considering the heat exchange process by inputting the convective coefficient (h) and the temperature of the surrounding fluid (T_∞_). To ensure no heat loss during the simulation, the default heat flux in the thermal insulation region was set to zero (q = 0). During mesh generation, the thermal conduction preset mesh was used, with mesh refinement applied in regions with high gradients, such as contact surfaces and heat sources, to ensure the accuracy of the simulation results. For convection simulations, boundary layer meshes were also added at the fluid-solid interface. Finally, by progressively refining the mesh and verifying mesh independence, it was ensured that the simulation results varied by less than 2%.

**7. EMW Absorption Measurement and RCS Simulation**

Based on the coaxial transmission/reflection mode, electromagnetic parameters were measured using a vector network analyzer (Agilent N5222A) in the frequency range of 2–18 GHz. The aerogels were cut into standard rings (ϕ_in_ = 3.04 mm, ϕ_out_ = 7.00 mm) using a custom-made cutter, placed in a mold and then a certain amount of molten paraffin was injected. After cooling and solidification, the composite specimens were demolded. The mass ratios of aerogel to paraffin were controlled at 1:99, 5:95 and 10:90, respectively, with three parallel samples prepared for each ratio to verify repeatability. The electromagnetic parameters include the complex permittivity (ε_r_ = εʹ-jεʺ) and the complex permeability (μ_r_ = μʹ-jμʺ), where εʹ (μʹ) and εʺ (μʺ) represent the material's ability to store and dissipate electromagnetic energy, respectively. Additionally, the dielectric loss tangent (Tanδ_ε_ = εʹ/εʺ) and magnetic loss tangent (Tanδ_μ_ = μʹ/μʺ) provide a measure of electromagnetic energy dissipation. Based on transmission line theory, the reflection loss (RL) for different absorber thicknesses can be determined using the following equation:

$Z_{in}\text{=}Z_{0}\sqrt{\frac{\mu_{r}}{\varepsilon_{r}}}\text{tan}\text{h}\text{[}\text{j}\frac{2\pi fd}{c}\sqrt{\mu_{r}\varepsilon_{r}}\text{]}$ (S1)

$\text{RL}\text{=20lg|}\frac{Z_{in}-Z_{0}}{Z_{in}+Z_{0}}\text{|}$ (S2)

where Z_in_, Z_0_, f, d and c represent the material impedance, air impedance, EMW frequency, material thickness and the speed of light in a vacuum, respectively.

Dielectric loss is a key mechanism for EMW absorption. According to Debye theory, Debye relaxation can be analyzed using the Cole-Cole curve:

$\left( \varepsilonʹ-\frac{\varepsilon_{s}-\varepsilon_{\infty}}{2} \right)^{2}+\left( \varepsilonʺ \right)^{2}={(\frac{\varepsilon_{s}-\varepsilon_{\infty}}{2})}^{2}$ (S3)

$\varepsilon^{'}=\varepsilon_{\infty}+\frac{1}{\tau}(\frac{\varepsilon^{''}}{\omega})$ (S4)

where ω is the angular frequency, ε_s_ is the low-frequency static permittivity, ε_∞_ is the high-frequency relative permittivity and τ is the relaxation time.

Magnetic loss contributes another part of the EMW absorption and can be indicated by the C_0_ value, which shows the mechanism of magnetic loss:

$C_{0}=\muʺ{(\muʹ)}^{-2}f^{-1}$ (S5)

When eddy current loss provides 100% of the magnetic loss, the C_0_ value does not change with frequency. If C_0_ changes, it is always accompanied by resonance.

The attenuation constant (α) can be used to measure the ability of the absorber to attenuate EMW energy:

$\text{α=}\frac{\sqrt{\text{2}}\text{πf}}{c}\text{×}\sqrt{\left( \mu\text{''}\varepsilon\text{''}\text{-}\mu\text{'}\varepsilon\text{'} \right)\text{+}\sqrt{\left( \mu\text{''}\varepsilon\text{''}\text{-}\mu\text{'}\varepsilon\text{'} \right)^{2}+\left( \mu\text{'}\varepsilon\text{''}\text{-}\mu\text{''}\varepsilon\text{'} \right)^{2}}}$ (S6)

Impedance matching is also a key factor in determining the EMW absorption performance of materials. To evaluate the impedance matching properties of the material, the delta-function method is introduced and the degree of impedance mismatch (|Δ|) can be described as follows:

$\mid\Delta\mid=\mid sinh^{2}(Kfd)-M\mid$ (S7)

$K=\frac{4\pi\sqrt{\varepsilon_{r}^{'}\mu_{r}^{'}}\sin(\frac{\delta_{\varepsilon}+\delta_{\mu}}{2})}{c\cdot\cos\delta_{\varepsilon}\cos\delta_{\mu}}$ (S8)

$M=\frac{4\mu^{'}cos\delta_{\varepsilon}\varepsilon^{'}\cos\delta_{\mu}}{{(\mu/cos\delta_{\varepsilon}-\varepsilon^{'}cos\delta_{\mu})}^{2}+{[\tan\frac{\delta_{\mu}-\delta_{\varepsilon}}{2}]}^{2}{(\mu^{'}\cos\delta_{\varepsilon}+\varepsilon^{'}\text{cos}\delta_{\mu})}^{2}}$ (S9)

where δ_ε_ = arctan(εʺ/εʹ) and δ_μ_ = arctan(μʺ/μʹ).

The radar cross-section (RCS) values were simulated using CST Studio Suite software. The simulation model consists of a double-layer square (200 × 200 mm), with a 2 mm thick absorbing layer on the top and a 1 mm thick perfect electric conductor (PEC) layer on the bottom. The simulation model is then placed on the X-O-Y plane and the EMW is incident in the negative Z direction, with θ as the incident angle, as shown in the following Fig. S1. Additionally, open boundary conditions were applied in all directions and the EMW frequency was set to 14.80 GHz.


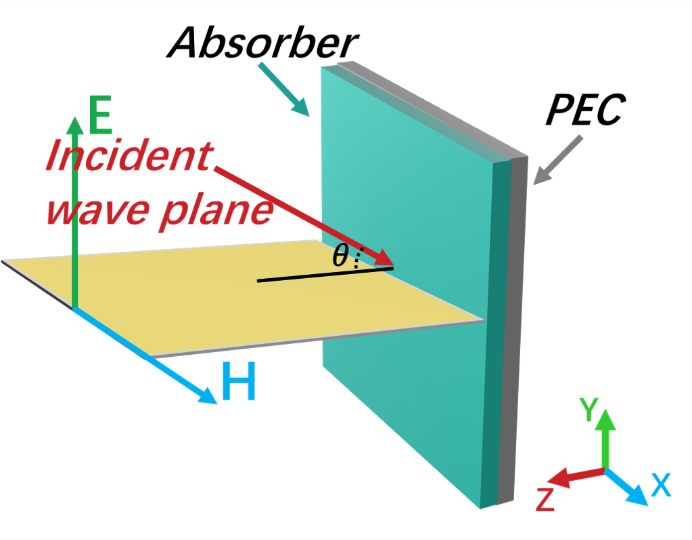


**Fig. S1.** Schematic of CST modeling.


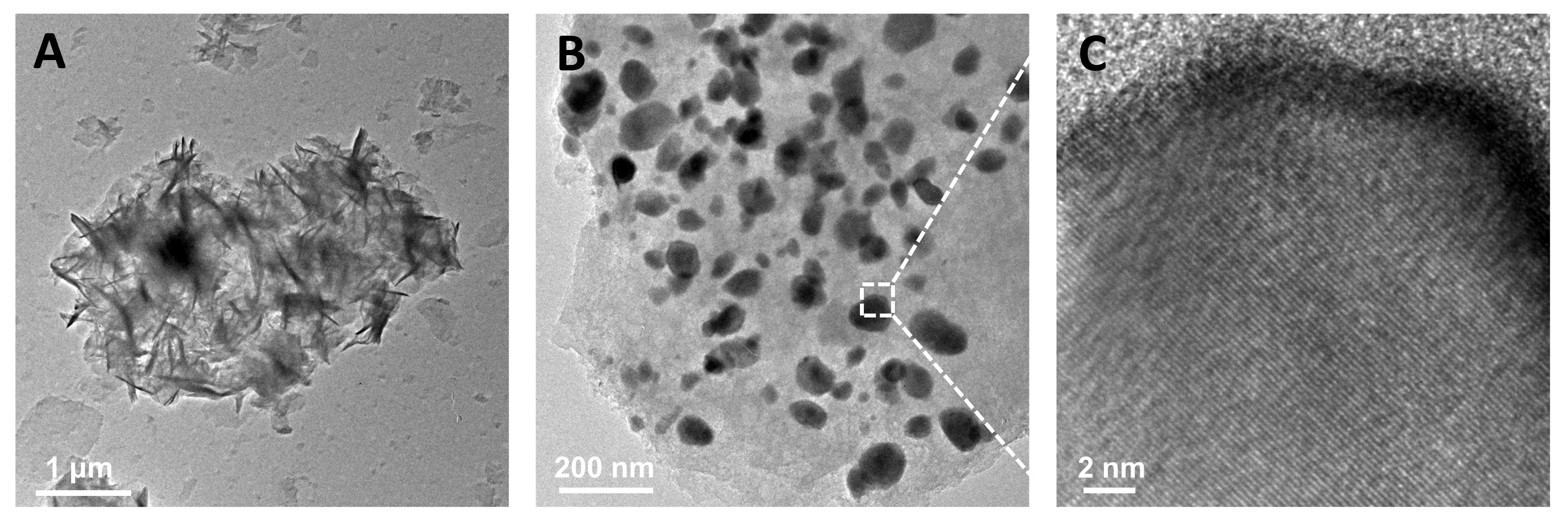


**Fig. S2.** TEM images of Ni-MOF-NH_2_ (A) and Ni-CCA (B, C).


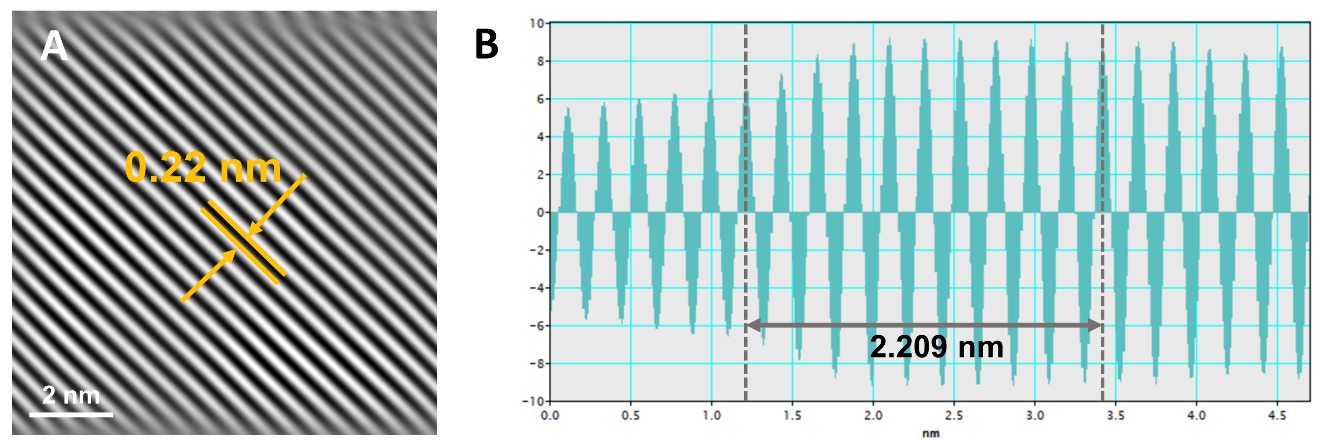


**Fig. S3.** IFFT image (A) and corresponding contrast intensity profile (B) of Ni-CCA.

Solid-state ^13^C nuclear magnetic resonance analysis (Fig. S4) elucidated the substitution patterns and substitution sites of phosphate groups on holocellulose. Holocellulose and PSU-holocellulose exhibited typical cellulose I (toric configuration) signals at 105.2 (C1), 89.8 (crystalline C4), 84.1 (amorphous C4), 73.5 (C3), 72.3 (C5), 66.0 (crystalline C6) and 63.5 ppm (amorphous C6) positions. After modification, PSU-holocellulose retained the basic framework of cellulose I (C4 double peak at 89.8/84.1 ppm; C1 at 105.2 ppm), confirming that the modification was localized on the surface and did not disrupt the cellulose I framework. The C6 spectral peak of PSU-holocellulose showed a 3.5 ppm downfield shift (65-68 ppm compared to 62.5 ppm of holocellulose), which was attributed to the electron attraction effect of the phosphate group (P=O) at the C6 hydroxyl position. This shift was quantitatively correlated with the hydroxyl substitution amount, confirming that the modification of C6 was achieved through the reduction of electronic density induced by the electron-withdrawing group. Meanwhile, the weakened signal intensity at C3 (73.5 ppm) indicated the presence of hydroxyl substitution.


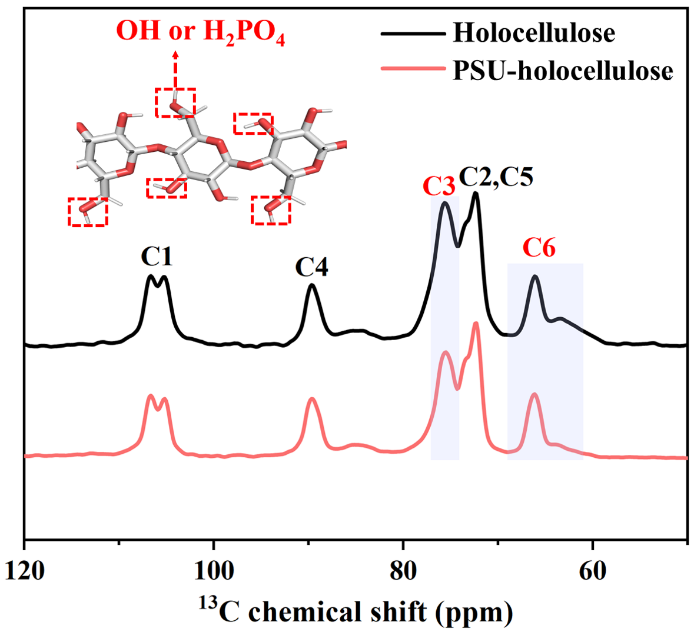


**Fig. S4.** NMR spectra of holocellulose and PSU-holocellulose.


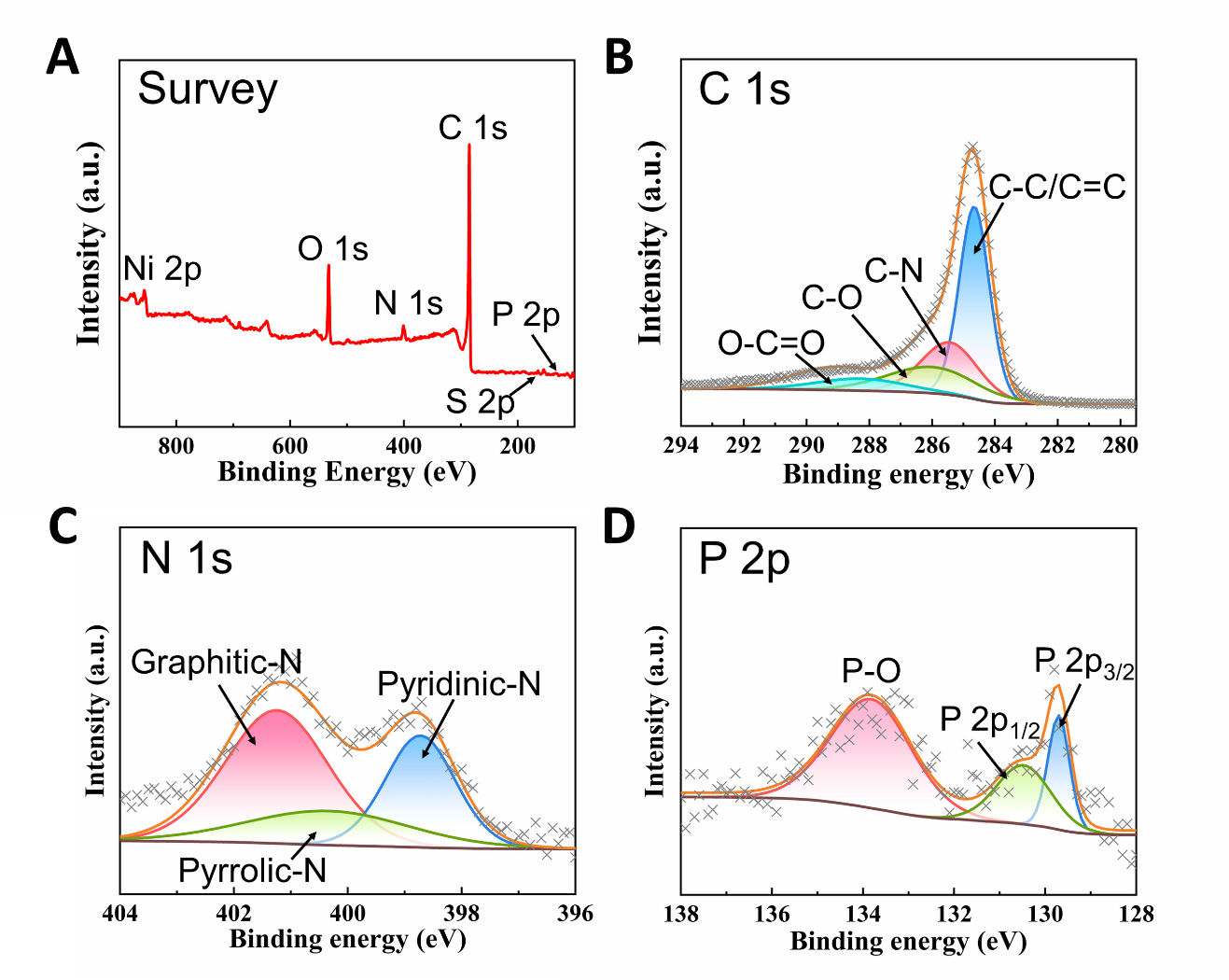


**Fig. S5.** XPS spectra of Ni-CCA: survey scan (A), C 1s (B), N 1s (C) and P 2p (D).


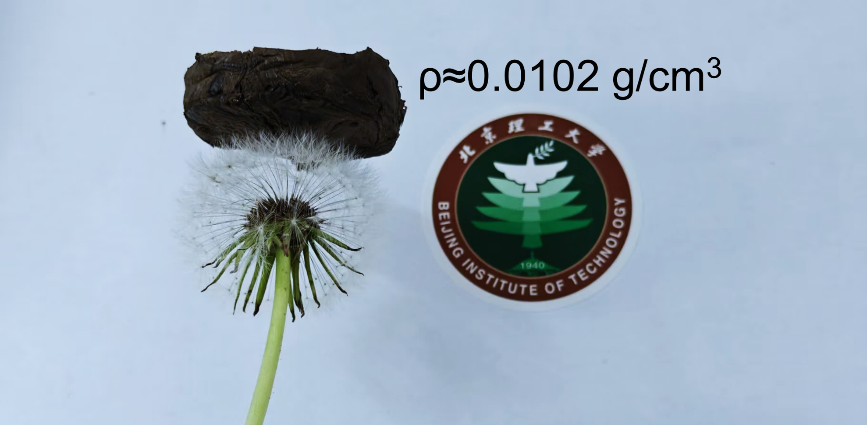


**Fig. S6.** Physical photo of Ni-CCA.


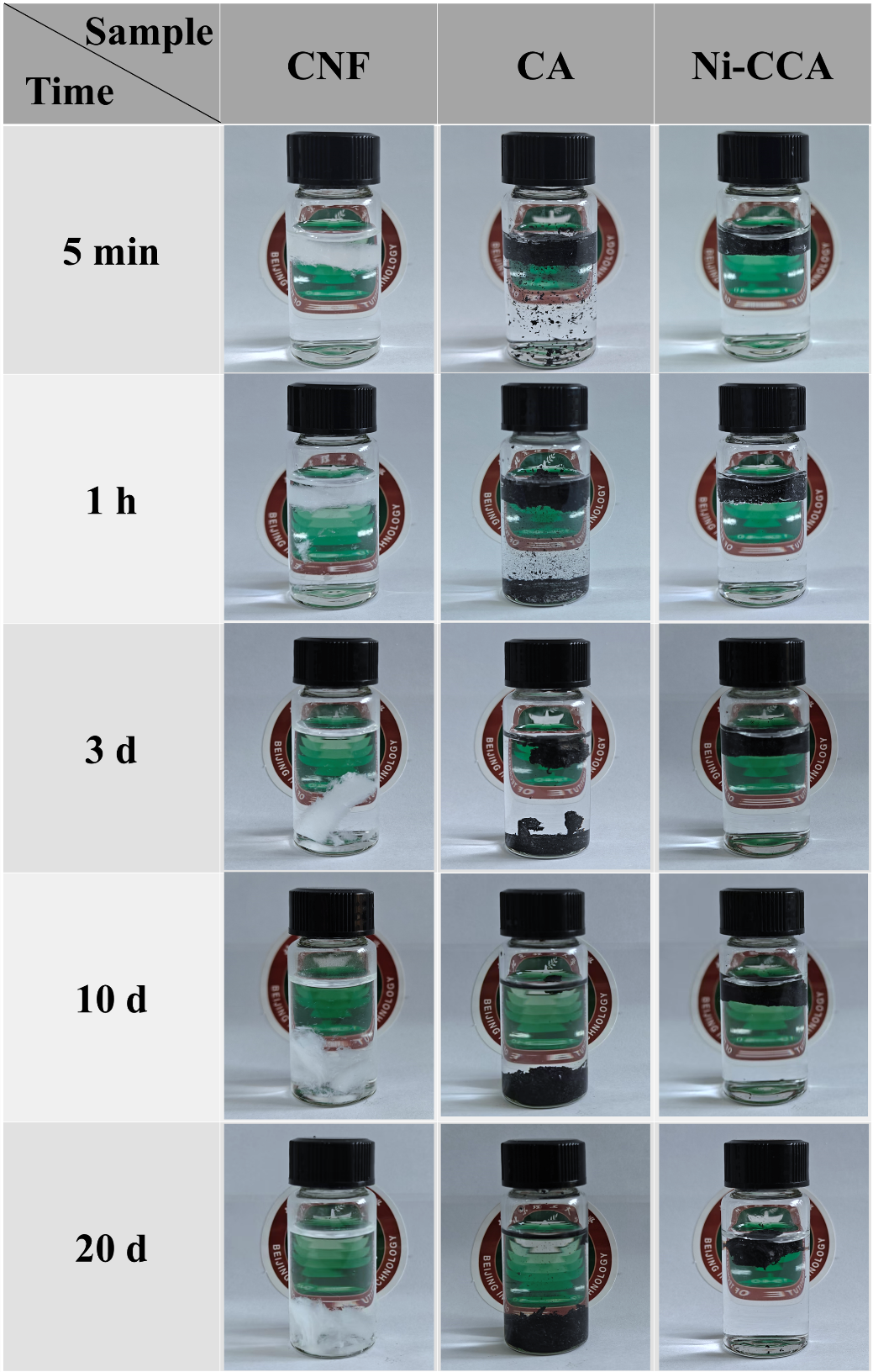


**Fig. S7.** Decomposition of CNF, CA and Ni-CCA in water from 5 min to 20 d.


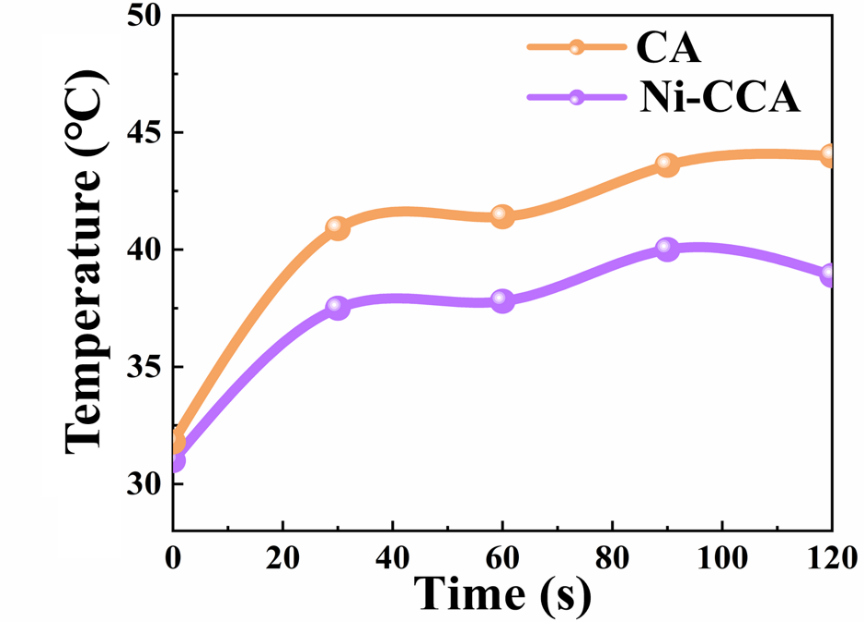


**Fig. S8.** Temperature curves of CA and Ni-CCA on 100℃ heating platform.


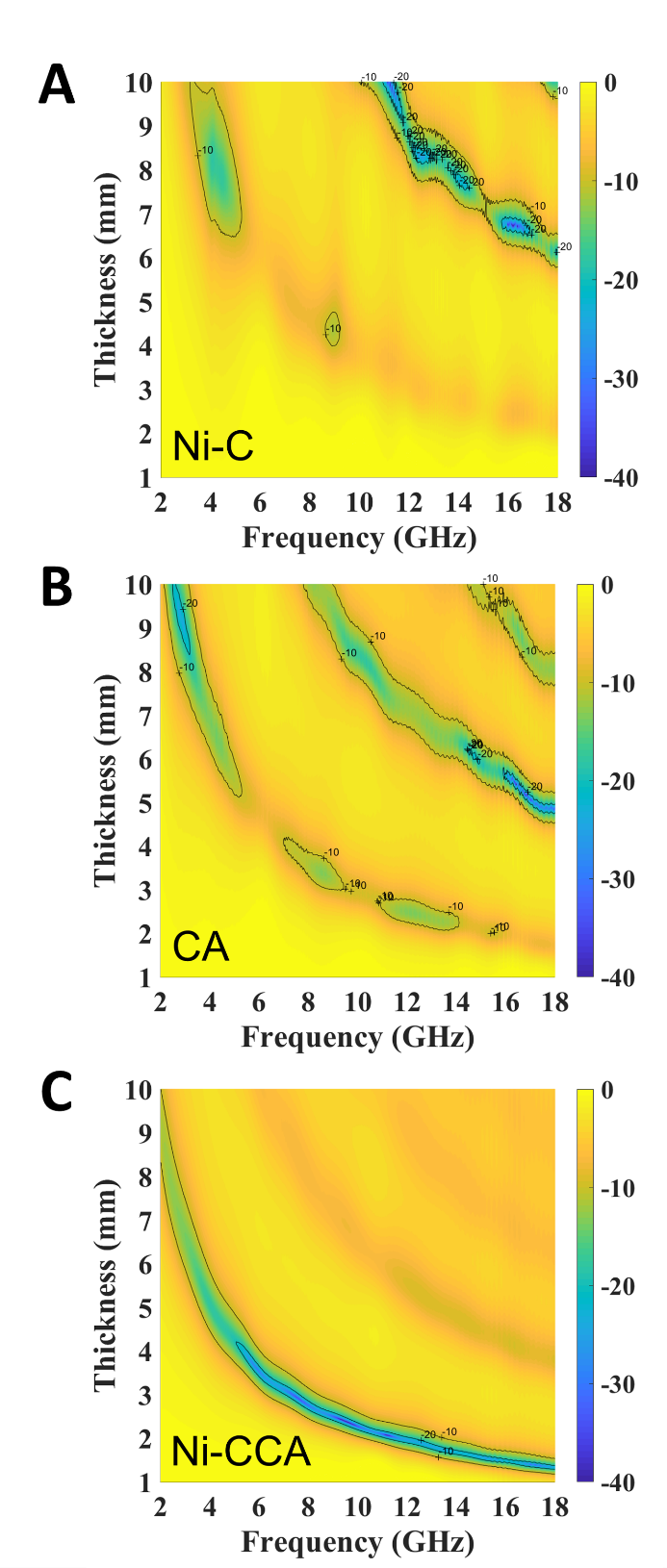


**Fig. S9.** Top view corresponding to RL plots of 5 wt.% loading Ni-C (A), CA (B) and Ni-CCA (C).

**Table S1.** Similar aerogels in recent years and their EMW absorption performance.

| **Sample** | **Component/key matrix** | **Filler loading (wt.%)** | **RL_min_ (dB)** | ***f*_RLmin_ (GHz)** | **T_RLmin_ (mm)** | **EAB (GHz)** | **T_EAB_ (mm)** | **Ref.** |
| --- | --- | --- | --- | --- | --- | --- | --- | --- |
| Ni, NiO | Metal oxides/porous carbon foam | 10 | -49.82 | 16.77 | 2.10 | 8.88 | 2.50 | [53] |
| MXene/Ni | MXene-Ni chains/bacterial cellulose | 15 | -31.90 | 17.52 | 1.00 | 2.30 | 1.00 | [54] |
| C/Ni-0.2/PPy | Ni-PPy/biomass carbon | 30 | -42.10 | 12.76 | 2.50 | 5.20 | 2.50 | [55] |
| C/Ni-0.6/PPy |  | 30 | -21.60 | 15.60 | 2.00 | 4.80 | 2.00 |  |
| Ni/N | Ni/N-doped carbon | 25 | -64.40 | 11.60 | 2.02 | 4.80 | 1.82 | [56] |
| Ni/C | Ni/BTC-derived carbon | 45 | -63.80 | 8.60 | 2.70 | 4.50 | 1.60 | [57] |
| CCMC/ZnO@Ni | ZnO@Ni-LDH/CMC-derived carbon | 40 | -64.00 | 13.90 | 2.00 | 4.90 | 2.00 | [58] |
| Ni@C-rGO | Metal@C microspheres/rGO | 20 | -53.64 | 7.92 | 4.10 | 6.64 | 2.55 | [59] |
| CoNi-NGA | CoNi/N-doped graphene | 8 | -43.84 | 8.31 | 3.00 | 4.24 | 1.80 | [60] |
| Ni/C@rGO | Ni/graphene foam | 15 | -22.70 | 18.0 | 1.30 | 5.20 | 1.50 | [61] |
| Ni-CCA | Ni_2_P/cellulose-derived carbon | 5 | -53.48 | 9.48 | 2.39 | 4.42 | 1.53 | This work |


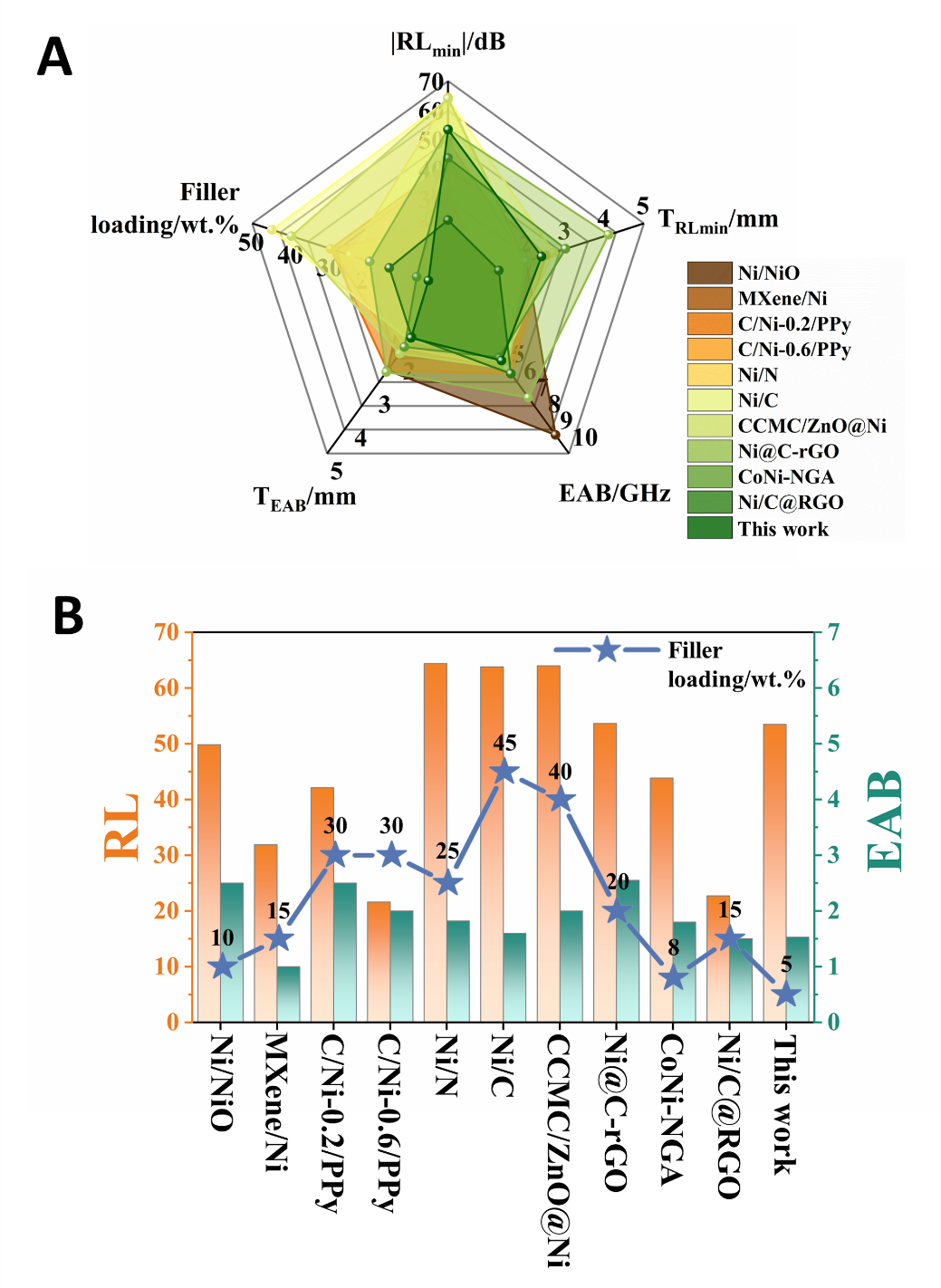


**Fig. S10.** Radar chart of EMW absorption performance (A) and RL-EAB comparison chart (B) of similar aerogels in recent years.


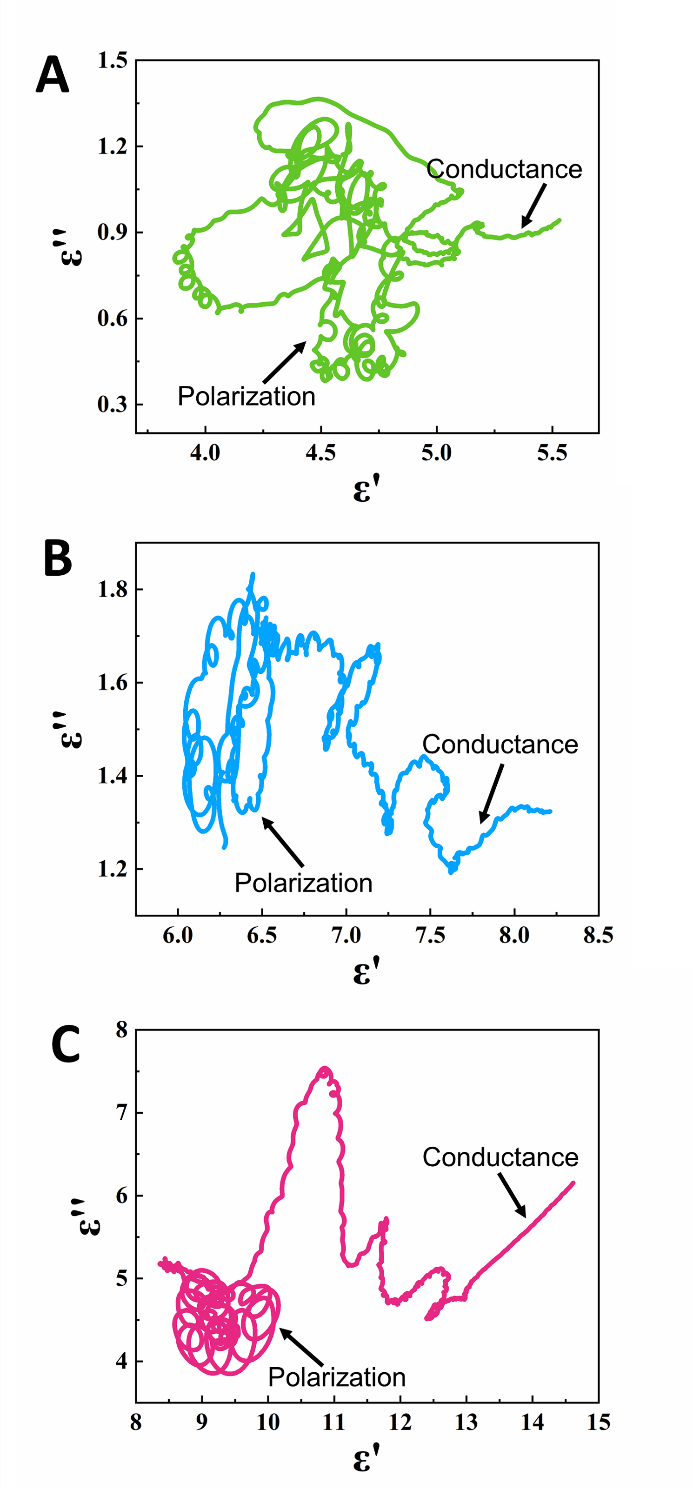


**Fig. S11.** Cole-Cole curves of 5 wt.% loading Ni-C (A), CA (B) and Ni-CCA (C).


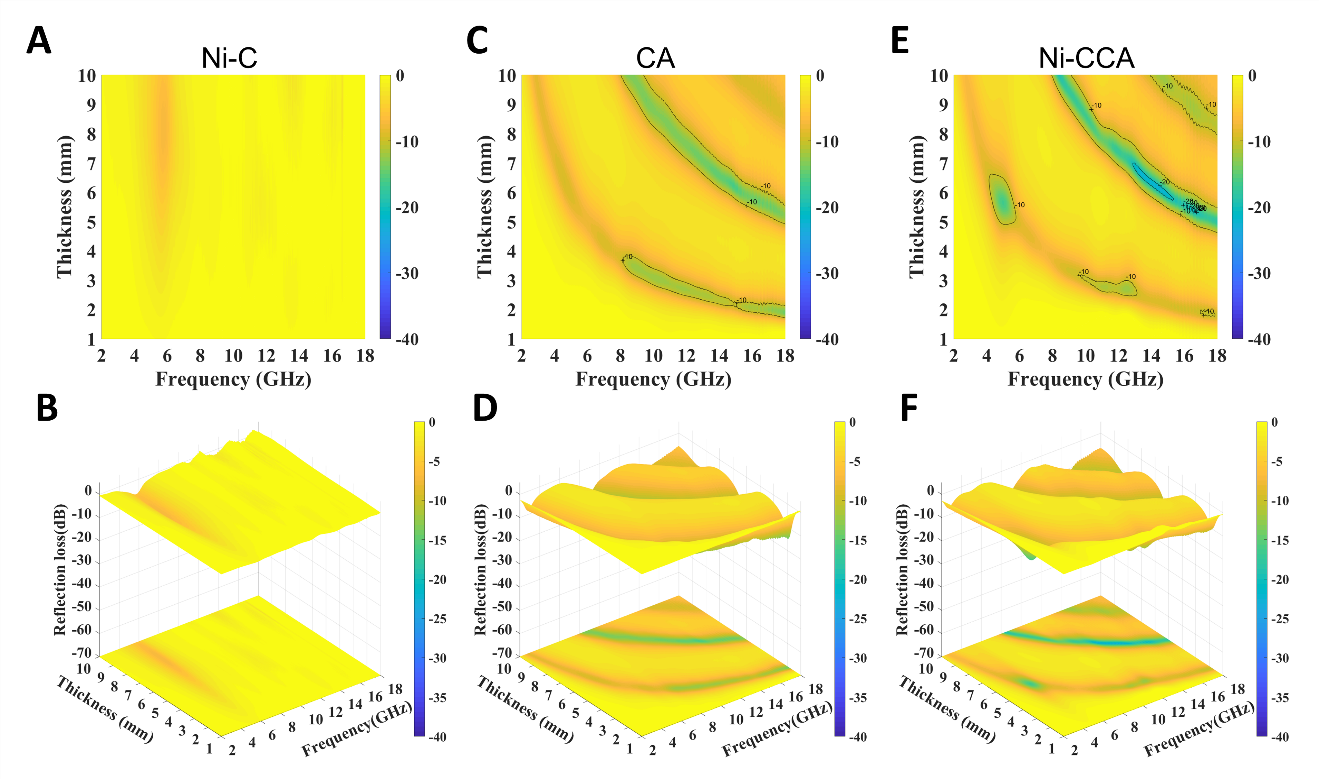


**Fig. S12.** RL plots of 1 wt.% loading Ni-C (A, B), CA (C, D), Ni-CCA (E, F) and corresponding top view.


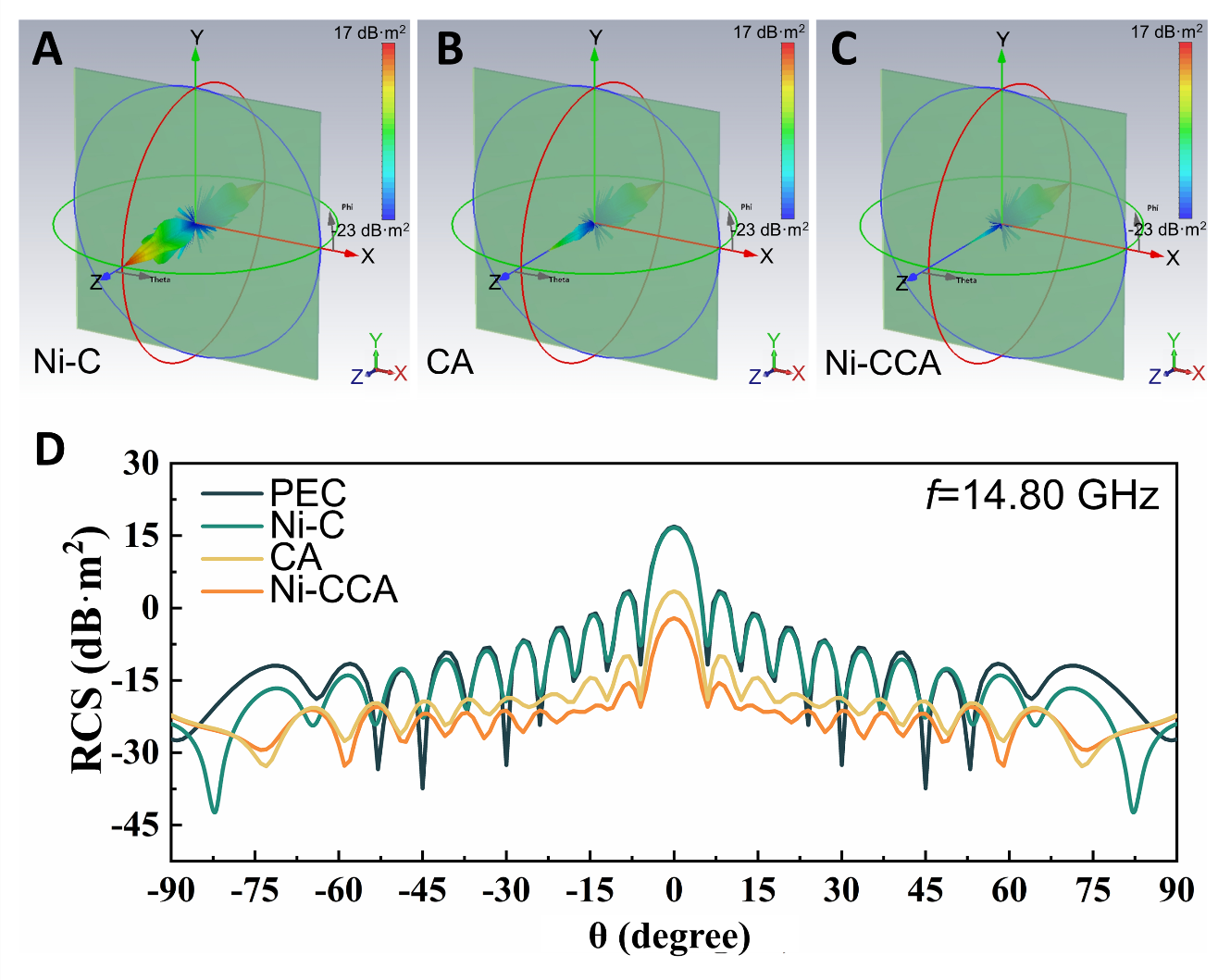


**Fig. S13.** RCS simulation of 1 wt.% loading Ni-C (A), CA (B) and Ni-CCA (C) and RCS curves (D).


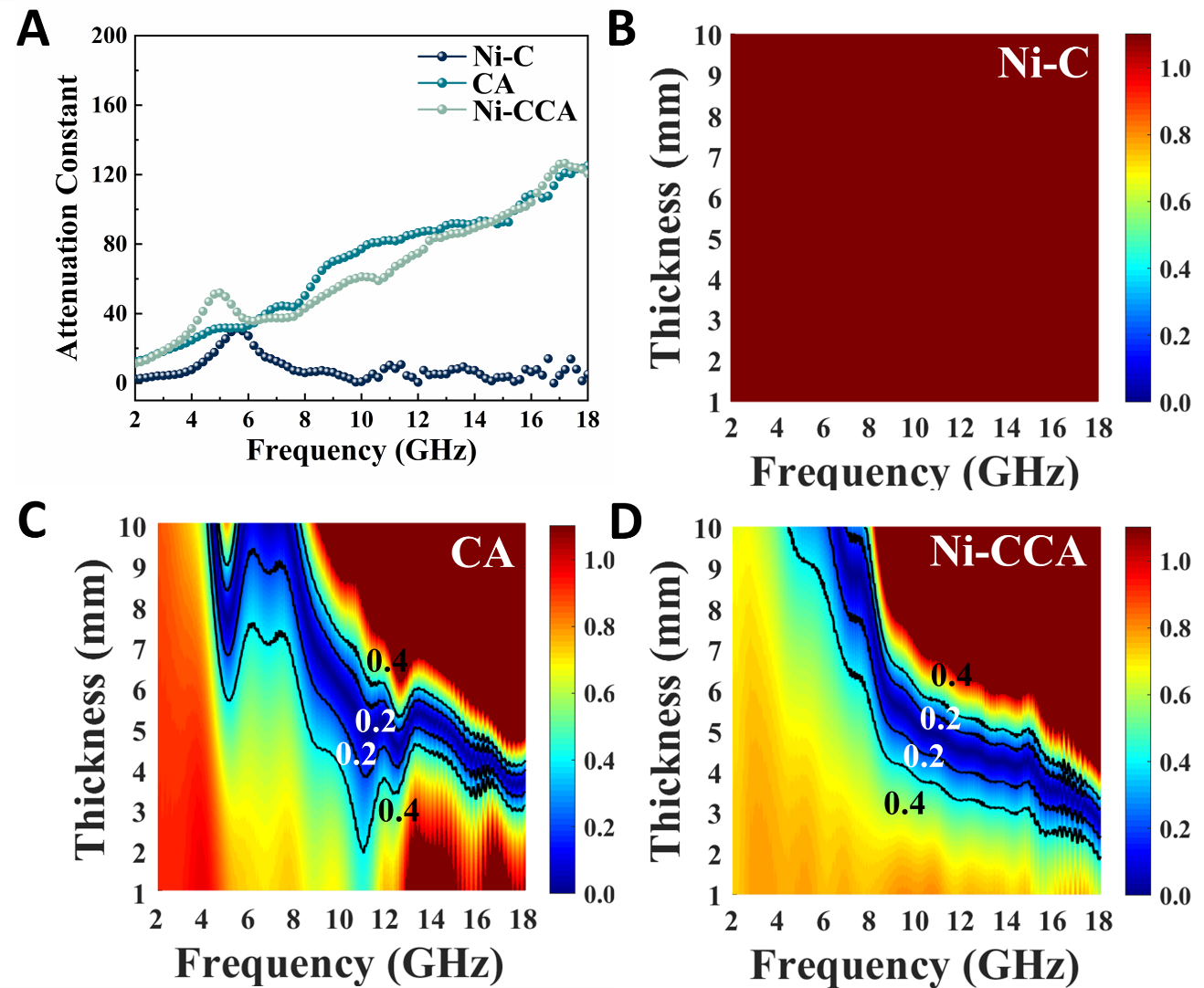


**Fig. S14.** Attenuation constants (A) and |Δ| diagram (B-D) of 1 wt.% loading Ni-C, CA and Ni-CCA.


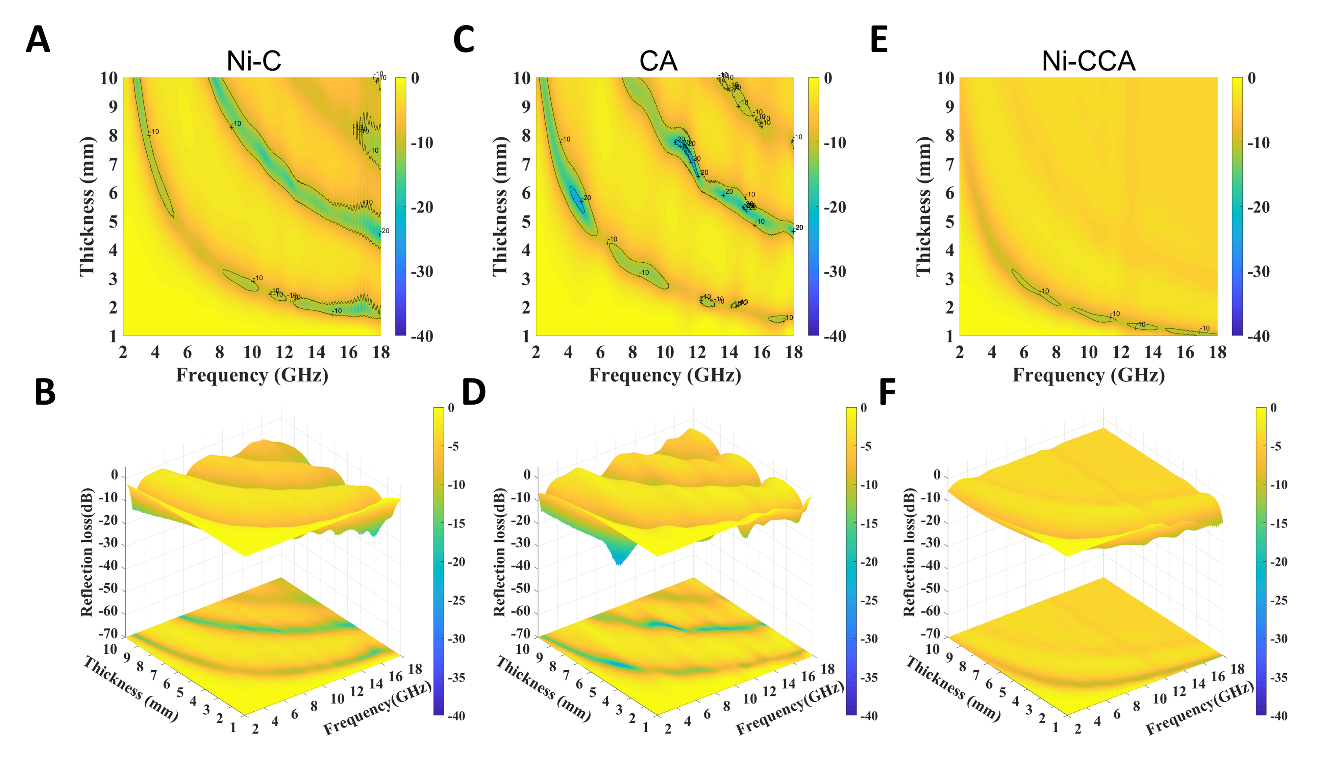


**Fig. S15.** RL plots of 10 wt.% loading Ni-C (A, B), CA (C, D), Ni-CCA (E, F) and corresponding top view.


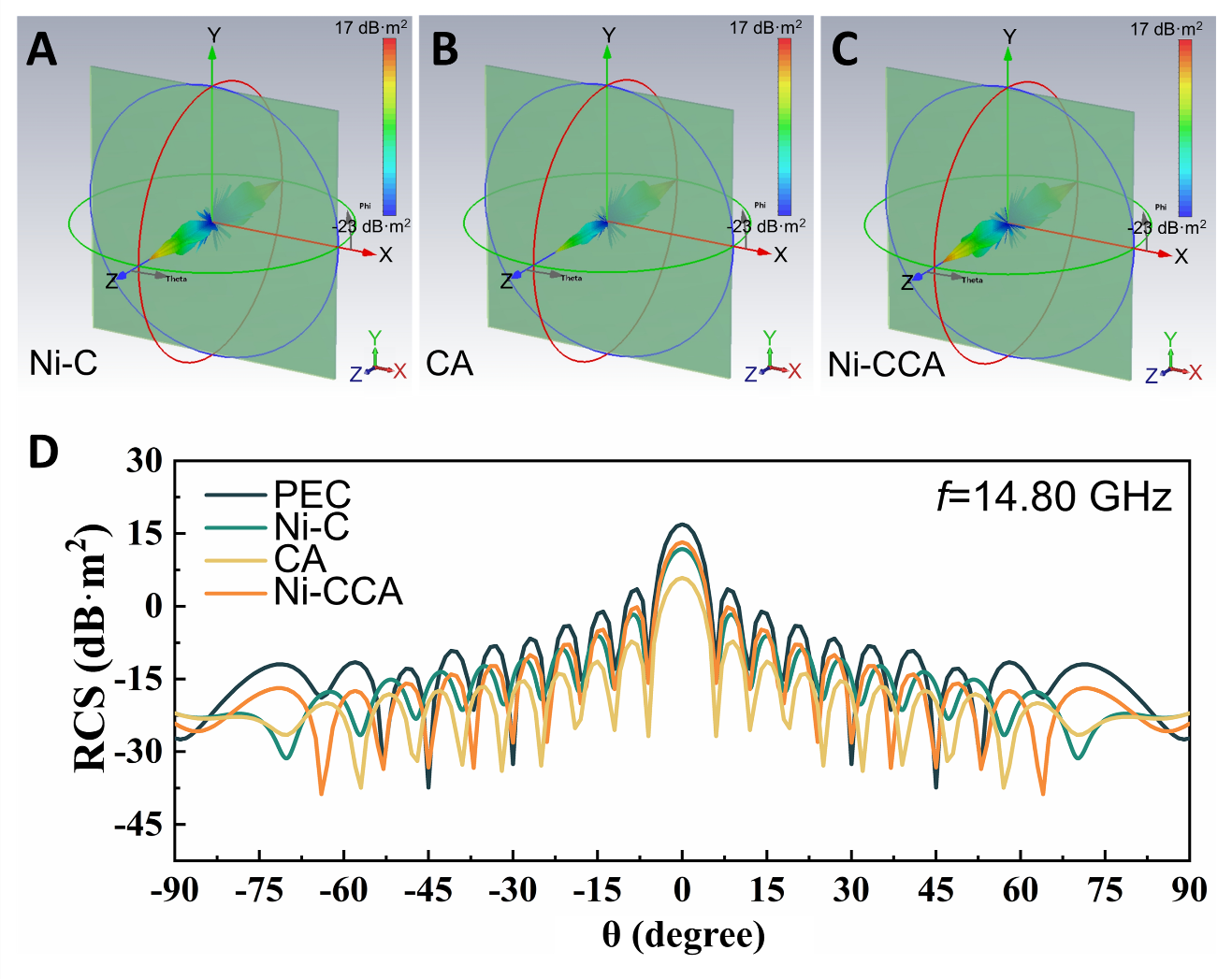


**Fig. S16.** RCS simulation of 10 wt.% loading Ni-C (a), CA (b) and Ni-CCA (c) and RCS curves (d).


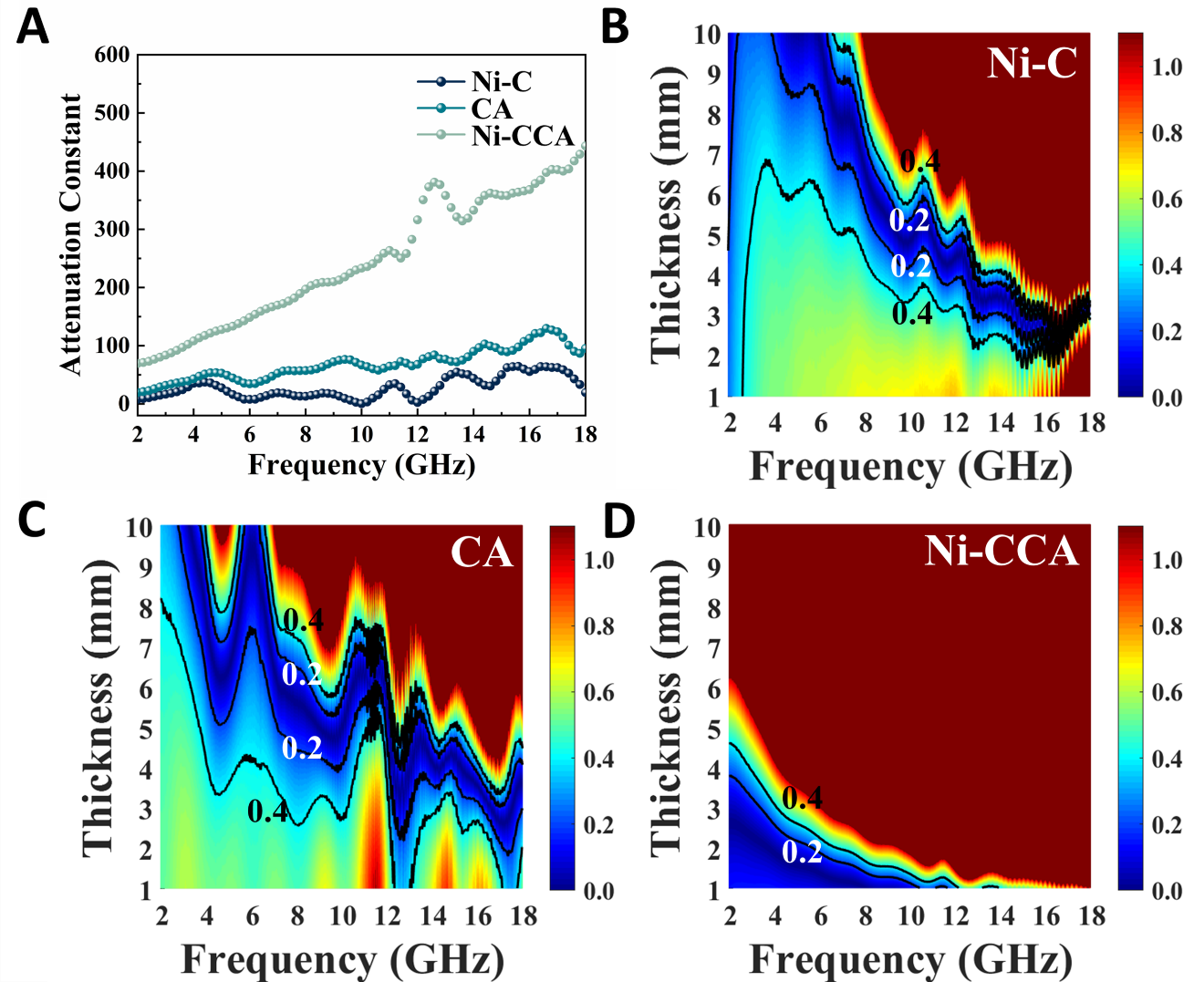


**Fig. S17.** Attenuation constants (A) and |Δ| diagram (B-D) of 10 wt.% loading Ni-C, CA and Ni-CCA.


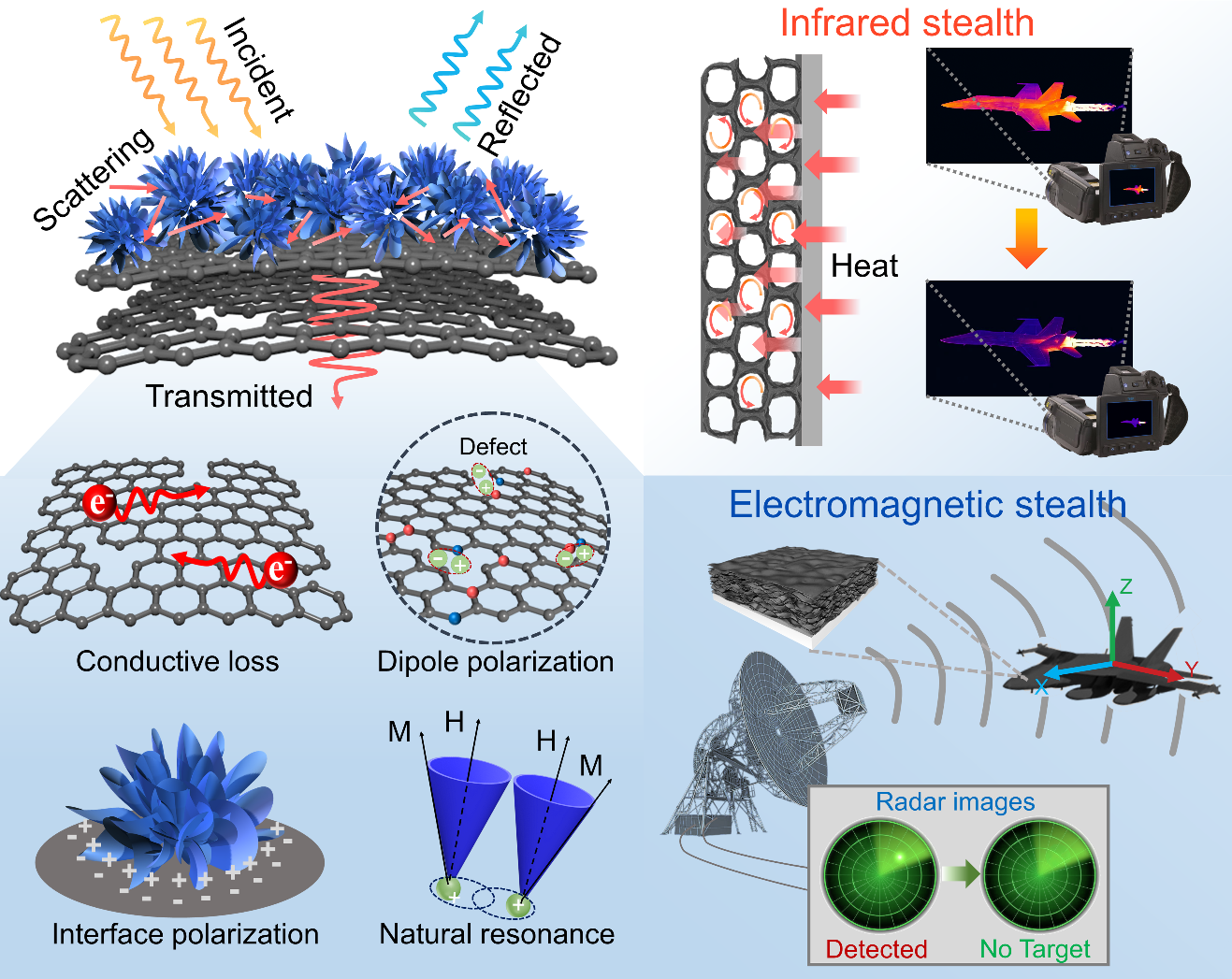


**Fig. S18.** The EMW absorption mechanism of Ni-CCA aerogel and its infrared/electromagnetic stealth application.
