# Supplementary figures and images for: Designing MOF-Cellulose Bio-Aerogels for Electromagnetic Management and Fire-Acoustic Safety
Source: Research (Wash D C). 2026 Feb 6;9:1111. doi: 10.34133/research.1111 (PMC12876565; doi:10.34133/research.1111)

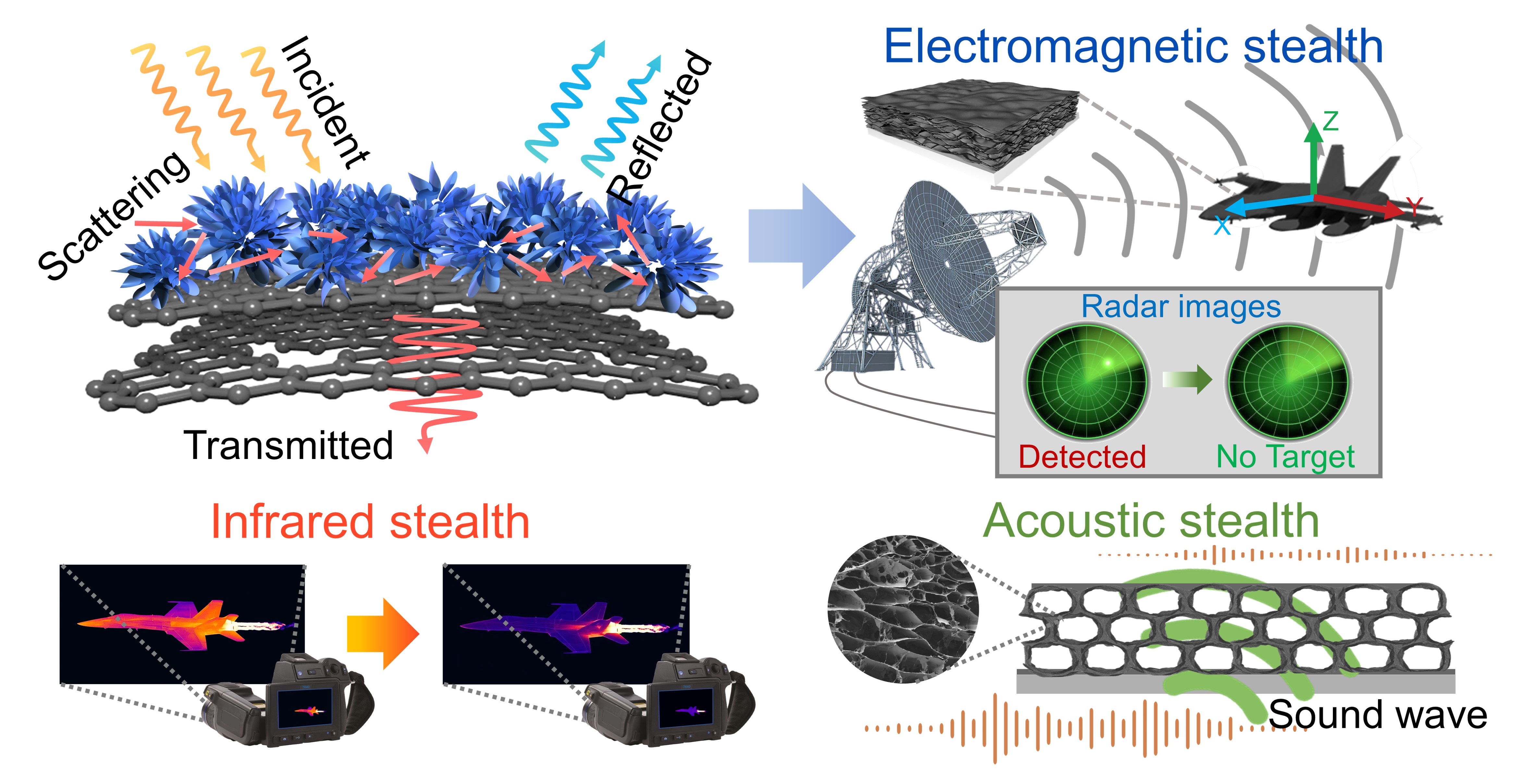

Supplement: Supplementary 1 — Figs. S1 to S18 Table S1 References [53–61] [file research.1111.f1.zip › ToC.jpeg]
